# Spotting Epidemic Keystones by *R*_0_ Sensitivity Analysis: High-Risk Stations in the Tokyo Metropolitan Area

**DOI:** 10.1371/journal.pone.0162406

**Published:** 2016-09-08

**Authors:** Kenta Yashima, Akira Sasaki

**Affiliations:** 1 Department of Evolutionary Studies of Biosystems, the Graduate University for Advanced Studies (SOKENDAI), Hayama, Kanagawa, Japan; 2 Meiji Institute for Advanced Study of Mathematical Sciences, Meiji University, Nakano, Tokyo, Japan; 3 Evolution and Ecology Program, International Institute for Applied Systems Analysis, Laxenburg, Austria; Hokkaido University Graduate School of Medicine, JAPAN

## Abstract

How can we identify the epidemiologically high-risk communities in a metapopulation network? The network centrality measure, which quantifies the relative importance of each location, is commonly utilized for this purpose. As the disease invasion condition is given from the basic reproductive ratio *R*_0_, we have introduced a novel centrality measure based on the sensitivity analysis of this *R*_0_ and shown its capability of revealing the characteristics that has been overlooked by the conventional centrality measures. The epidemic dynamics over the commute network of the Tokyo metropolitan area is theoretically analyzed by using this centrality measure. We found that, the impact of countermeasures at the largest station is more than 1,000 times stronger compare to that at the second largest station, even though the population sizes are only around 1.5 times larger. Furthermore, the effect of countermeasures at every station is strongly dependent on the existence and the number of commuters to this largest station. It is well known that the hubs are the most influential nodes, however, our analysis shows that only the largest among the network plays an extraordinary role. Lastly, we also found that, the location that is important for the prevention of disease invasion does not necessarily match the location that is important for reducing the number of infected.

## Introduction

Theoretical analyses based on mathematical models have been shown to be useful for planning and evaluating various intervention strategies against infectious disease [1,2]. For instance, the strategies for containing an emerging influenza in the Southeast Asia [3,4] and the mitigation strategies for an influenza pandemic in the United States, the United Kingdom [5–7] and also in the Tokyo metropolitan area [8–12] have been studied taking into consideration of the actual population structure and movement data. These geographically explicit models enable us to create practical and specific intervention policies. However, these require a comprehensive knowledge of the individuals’ mobility pattern and their person-to-person contacts, which are generally difficult to obtain. The properties of the metapopulation network model are suitable for modeling mobility patterns in the metropolitan area, where inter-district flows (i.e., commuter flow between train stations) are well known but intra-district flows (i.e., local movements around the train station) are not. On this basis, we have formulated the commute network model of the Tokyo metropolitan area as a metapopulation network model, such that each station corresponds to the node and commuting flow corresponds to the edge. Here, the infection is assumed to occur locally within the residence area or the workplace and spreads out globally across the metropolitan area through the individuals’ commuting. This formulation is particularly suitable for modeling epidemic dynamics of pathogens transmitted by fecal-oral infection, such as norovirus and enteroviruses, where the infections occur much more frequently in the home, the workplace, and nearby restaurants [13,14], than in the course of commuting.

In the theory of networks it is well known that, when the number of connections for each node (i.e., degree) follows a heavy-tailed distribution, it has a notable effect on the epidemic dynamics, and shown to reduce the disease invasion threshold in the transmission rate, enabling an epidemic to occur even for a disease with very weak transmission ability [15–21]. In such case, an epidemic cannot be prevented efficiently by random vaccination/quarantine, and instead countermeasure such as targeted immunization is required and has shown to be highly effective [22–29]. The application of these strategies requires information on each node about its relative importance within the network. Several network centrality measures [30] have been utilized in identifying these influential nodes by quantifying its relative importance: degree centrality [22–24], betweenness centrality [26,27], k-coreness [28], and dynamic influence [29].

**Fig 1:**
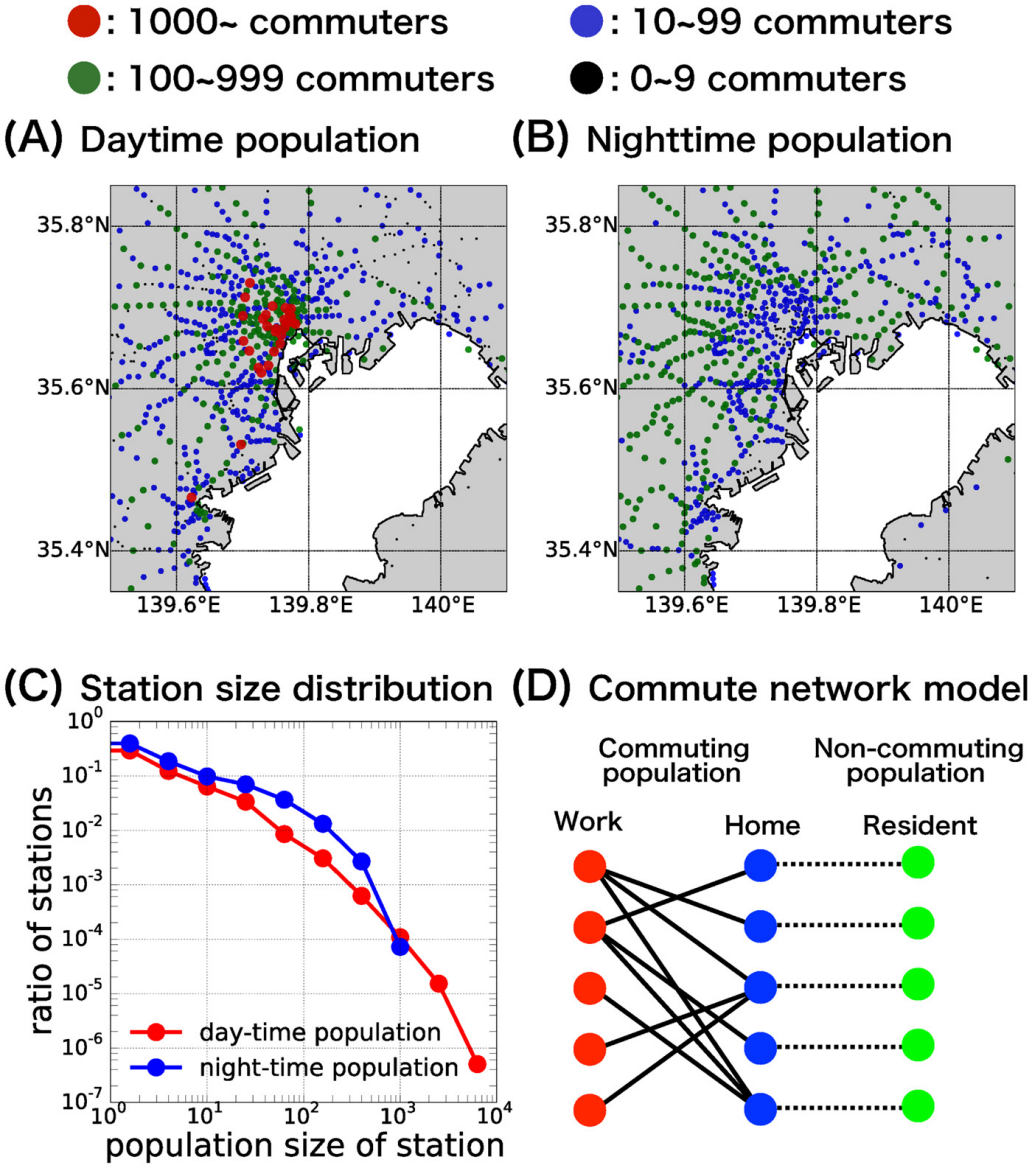
Commute network model of the Tokyo metropolitan area. Geographical distribution of daytime working/studying population (A) and nighttime residing population (B) at each station. Each dot corresponds to a single station, with the color indicating its population size. The red colored stations near the center of (A) corresponds to the inner urban area of the Tokyo metropolitan area, which include the largest working station Shinjuku station, the second largest Tokyo station, and the third largest Shibuya station; the two red stations in the lower left are the Kawasaki and Yokohama stations. The longitude and latitude of each station were acquired from the Station Database [http://www.ekidata.jp]. (C) Population size distribution of the daytime working/studying population (red line) and the nighttime residing population (blue line) at each station. (D) Illustration of the commute network model. Each station has a working/studying area (daytime “work population”, red circles) and a residing area (nighttime “home population”, blue circles) connected by a commuting flow (“commuting population”). The non-commuting population at each station (“resident population”, green circles) is connected to the corresponding home population.

Various transportation networks show heavy-tailed distributions for both the connectivity patterns (i.e., degree) and for the sizes of traffic flows [21,31]. This heavy-tailed distributions in the population sizes is also observed in the commuting network of the Tokyo metropolitan area (Fig 1A–1C) [32]. Therefore, identification and evaluation of epidemiologically high-risk communities (i.e., nodes) using the centrality measure is essential for the disease prevention in a metropolitan area. Most of previous researches have utilized the centrality measures based solely on the static topological structure of the network [22–24,26–28]. Since the epidemic is a dynamic process, it is reasonable to adopt these dynamical aspects in the centrality measure itself to get improved evaluation for the relative importance of each node in epidemics. The basic reproductive ratio *R*_0_ [1], is generally used as a criterion for the invasion of disease: if *R*_0_ > 1, invasion of disease occurs and if *R*_0_ < 1, the disease fails to invade. For this reason, we have introduced a novel centrality measure (called “*R*_0_ centrality”) that utilizes this *R*_0_ as a control objective, which gives a straightforward estimation of the effect of countermeasures. By using this centrality measure, markedly strong influence of the largest subpopulation has been revealed: only the largest among a number of sufficiently large subpopulation shows prominent role in preventing the invasion of disease. To the best of our knowledge, none of the conventional centrality measures showed this feature. The epidemic dynamics over the commute network of the Tokyo metropolitan area is analyzed using this centrality measure. The *R*_0_ is obtained by using the next generation matrix method [33], and then by applying a sensitivity analysis [34,35] the marginal change of *R*_0_ is calculated when the countermeasures are applied. This enabled us to define the centrality measure for each local population, which estimates its epidemiological influence (i.e., a countermeasure is more effective in reducing *R*_0_, when applied in a local population with a higher *R*_0_ -centrality).

## Methods

### Commute network model

The commute network data of the Tokyo metropolitan area were obtained from the 10^th^ Urban Transportation Census Report (UTC) [36], a questionnaire survey conducted by the Japanese Ministry of Land, Infrastructure, Transport and Tourism, which is intended to provide basic data for public transportation policies within the metropolitan area. The UTC data enabled us to trace the daily commute movements of 139,841 individual commuters (approximately 0.4% of the total number of residents in the Tokyo metropolitan area) between their residing stations at nighttime and working/studying stations at daytime. The geographical distributions and its population size of the daytime population and the nighttime population of the Tokyo metropolitan area are given in Fig 1A and 1B. The numbers are from the collected questionnaires of the UTC; therefore, when considering the actual population, the number of commuters should be multiplied by a factor of approximately 100 (as not all of the individuals are commuting). Hereafter, the term “population size” refers to the UTC sample data and not to the actual population. The population size distribution of the daytime working/studying population and the nighttime residing population at each station are given in Fig 1C. The largest daytime population size exceeded 5,000 commuters (working/studying area of Shinjuku station with the population size of 5,411); whereas the largest nighttime population size was lower than 1,000 (residing area of Oizumi-gakuen station with the population size of 853). Using this UTC data, we are able to follow the intra-regional commuting flow within the Tokyo metropolitan area. Further details about the utilized UTC data were presented in our previous paper [32].

A model of the commute network was formulated as a bipartite metapopulation network (see Fig 1D for the schematic diagram), with two types of populations accompanying each station: work/study area (the “*work population*”, denoted by red circles in Fig 1D) and residence area (the “*home population*”, denoted by blue circles in Fig 1D). A home population is connected to work populations but not to the other home populations; a work population is connected only to home populations (connections are denoted by solid lines in Fig 1D). Each commuter is assumed to travel daily from his/her home population to his/her work population using a commuter train, stay at the work population during daytime and return to the home population using the same commuter train and stay there during nighttime. The importance of such recurrent commuting patterns to the epidemic dynamics has been reported previously, and these patterns appear to be quite different from the results of simple random mobility patterns [37–40]. Let $N_{ij}^{C}$ be the number of individuals commuting from their residence area at station *i* (i.e., the *i*-th home population) to their work/study area at station *j* (i.e., the *j*-th work population). From this, we define $N_{i}^{H}=\overset{}{\underset{}{\sum_{j=1}^{M}N_{ij}^{C}}},$ $N_{j}^{W}=\Sigma_{i=1}^{M}N_{ij}^{C}$, and $N^{C}=\Sigma_{i=1}^{M}\Sigma_{j=1}^{M}N_{ij}^{C}$ (*M* = 1,435: the total number of stations in the Tokyo metropolitan area), which respectively gives the number of commuters that use *i*-th station as the home population, the number of commuters that use *j*-th station as the work population, and the total number of commuters in the metropolitan area.

As the UTC data only contain information about the commuting individuals, we have assumed, in order to incorporate the effect of non-commuting individuals, that the number of non-commuting individuals who reside and do not commute at each station (the “*resident population*”, denoted by green circles and connected to the corresponding home population with a black dotted line in Fig 1D) are proportional to the number of residing and commuting individuals at each station. That is to say, the number of non-commuting individuals at the *i*-th resident population is given as $N_{i}^{R}=rN_{i}^{H}$ (*r*: the ratio of the number of non-commuting residents to that of commuting residents). For simplicity, we further assumed that this proportionality factor *r* is the same for all the populations. The total number of non-commuting individuals is thus given by $N^{R}=\overset{}{\underset{}{\sum_{i=1}^{M}N_{i}^{R}=rN^{C}}},$ and the total population of the metropolitan area is given by *N* = *N*^*R*^ + *N*^*C*^ = (*r* + 1) *N*^*C*^. In summary, the *k*-th station is characterized by three population sizes (Fig 1D): the number $N_{k}^{W}$ of commuters who come to the *k*-th station to work/study (work population), the number $N_{k}^{H}$ of commuting residents (home population), and the number $N_{k}^{R}$ of non-commuting residents (resident population). Because we did not have the relevant data for the non-commuting individuals, the ratio *r* was left as an adjustable parameter. In this study we used *r* = 1, i.e., the number of non-commuting individuals were the same as the number of the commuting individuals at each station. There was no significant difference in the epidemic dynamics when *r* was varied over a realistic range. Further discussion on the adjustment of *r* is given in S2 File in relation to the final size of epidemic (S1 Fig).

### Epidemic model

The spread of infectious disease over the commute network of the Tokyo metropolitan area was modeled in the following way. All individuals were classified into susceptible, infectious, or recovered state (SIR model) [1]. Then, the number of non-commuting individuals in the *i*-th resident population was decomposed into $N_{i}^{R}=S_{i}^{R}\left(t\right)+I_{i}^{R}\left(t\right)+R_{i}^{R}\left(t\right),$ where the number of susceptible, infectious, and recovered individuals in the non-commuting populations are denoted by $S_{i}^{R}\left(t\right)$, $I_{i}^{R}\left(t\right)$ and $R_{i}^{R}\left(t\right)$, respectively. In the same vein, the number of commuting individuals who reside in the *i*-th home population at night and commute to the *j*-th work population at daytime, $N_{ij}^{C},$ was decomposed into $N_{ij}^{C}=S_{ij}^{C}\left(t\right)+I_{ij}^{C}\left(t\right)+R_{ij}^{C}\left(t\right)$, where the number of individuals in each state for the commuting populations are denoted as $S_{ij}^{C}\left(t\right)$, $I_{ij}^{C}\left(t\right)$, and $R_{ij}^{C}\left(t\right)$. The infection was assumed to occur in the home, work, and resident populations, whereas infection during the commuting process was neglected (the limitation due to this assumption will be given in the discussion). The following system of ordinary differential equations describes the epidemic process:

$$\frac{dS_{i}^{R}\left(t\right)}{dt}=-\beta \left(2I_{i}^{R}\left(t\right)+\Sigma_{k}I_{ik}^{C}\left(t\right)\right)S_{i}^{R}\left(t\right)$$ (1)

$$\frac{dI_{i}^{R}\left(t\right)}{dt}=\beta \left(2I_{i}^{R}\left(t\right)+\Sigma_{k}I_{ik}^{C}\left(t\right)\right)S_{i}^{R}\left(t\right)-\gamma I_{i}^{R}\left(t\right)$$ (2)

$$\frac{dR_{i}^{R}\left(t\right)}{dt}=\gamma I_{i}^{R}\left(t\right)$$ (3)

$$\frac{dS_{ij}^{C}\left(t\right)}{dt}=-\beta \left(I_{i}^{R}\left(t\right)+\Sigma_{k}I_{ik}^{C}\left(t\right)+\Sigma_{k}I_{kj}^{C}\left(t\right)\right)S_{ij}^{C}\left(t\right)$$ (4)

$$\frac{dI_{ij}^{C}\left(t\right)}{dt}=\beta \left(I_{i}^{R}\left(t\right)+\Sigma_{k}I_{ik}^{C}\left(t\right)+\Sigma_{k}I_{kj}^{C}\left(t\right)\right)S_{ij}^{C}\left(t\right)-\gamma I_{ij}^{C}\left(t\right)$$ (5)

$$\frac{dR_{ij}^{C}\left(t\right)}{dt}=\gamma I_{ij}^{C}\left(t\right)$$ (6)

Here, *β* is the infection rate and *γ* is the recovery rate, both of which are assumed to be the same for every local population. In this study, the average duration of the infected state was fixed as 2 days; hence, a recovery rate of *γ* = 0.5 was used in all the calculations. It was also assumed that commuting individuals spend the day at their work population and night at their home population, whereas non-commuting individuals spend both day and night at their resident population. Here, the working district and the district of residence at the same station are assumed to be in geographically distinct locations, therefore the disease transmission between the working commuters and the non-commuting residents at the same station is neglected. The first term in parentheses on the right hand sides of Eqs (1), (2), (4) and (5) gives the force of infection from the infected non-commuting population at the *i*-th resident population, where factor 2 comes from the fact that non-commuting population spend both day and night at their station (i.e., we assumed that day and night are equal in duration). The second term in parentheses of Eqs (1), (2), (4) and (5) gives the force of infection from the infected commuters who reside in the *i*-th home population at nighttime. The last term in parentheses of Eqs (4) and (5) gives the force of infection from the infected commuters who stay with the *j*-th work population at daytime. The recovery process is denoted in the final terms of Eqs (2), (3), (5) and (6).

The following section describes how the basic reproductive ratio *R*_0_, which sets the condition for the disease invasion, was calculated. Once defined, the sensitivity of *R*_0_ to countermeasures applied at each local population can be analyzed. The impact of disease spread was quantified as the final size of the epidemic (the fraction of individuals within the population who eventually become infected), against which the effectiveness of intervention strategies could be evaluated. The formulation and numerical method for calculating the final size of the epidemic are given in S2 File.

### Basic reproductive ratio *R*_0_

The condition for the invasion of an infectious disease can be assessed explicitly by using the basic reproductive ratio *R*_0_, which is defined as the mean number of secondary infections in a completely susceptible population from a single initially infectious individual during its entire period of infectiousness [1,41–43]. Given this definition, the invasion condition of an infectious disease can be denoted as *R*_0_ > 1. The final size of an epidemic, *Ψ*, is positive if *R*_0_ > 1 or zero if *R*_0_ < 1. For a single well-mixed population model, the calculation of *R*_0_ is straightforward: *R*_0_ = [infection rate] × [number of susceptible individuals]/[recovery rate]. However, a structured population model, such as our metapopulation model, requires a further extension of this definition because the initial infectious population will be distributed over the local populations, and the mean number of secondary infections will depend on the location of the initially infected host. To take these points into consideration, *R*_0_ is defined in structured population models as the dominant eigenvalue of the next generation matrix ***L*** [33,43,44].

The next generation matrix ***L*** can be formulated as the following 3 × 3 block matrix. This can be done by linearizing the epidemic dynamic Eqs (1)–(6) with respect to the number of infected individuals, or by replacing $S_{i}^{R}\left(t\right)$ by $N_{i}^{R}$ and $S_{ij}^{C}\left(t\right)$ by $N_{ij}^{C}$, and by applying the integral forms of Eqs (2) and (5):

$$L=\frac{\beta }{\gamma }\left[\begin{array}{ccc} T_{RR} & T_{RH} & 0\\ T_{HR} & T_{HH} & T_{HW}\\ T_{WR} & T_{WH} & T_{WW} \end{array}\right]$$ (7)

The detailed expression and derivation of ***L*** is given in S1 File. Here each block element in ***L*** corresponds to the type of population: the non-commuting resident populations (R), the commuting home populations (H), and the commuting work populations (W). The block element ***T***_*mn*_ is a *M* × *M* matrix (where *M* is the total number of stations) denoting the transmission from type *n* populations to type *m* populations (*m*,*n* ∈ {*R*, *H*, *W*}), see S1 File for detailed expressions for the values of *T*_*mn*_. The basic reproductive ratio is given by *R*_0_ = *ρ*(***L***), where *ρ*(***L***)gives the dominant eigenvalue of the next generation matrix ***L*** [33,43,44]. As seen in Eq (7), the block matrix ***L*** is made up of the factor *ρ*_0_ ≡ *β*/*γ* (the “epidemiological factor”) defined only by the infection rate and recovery rate, and the matrix describing the host population structure (the “host population structure matrix”). By denoting the dominant eigenvalue of the host population structure matrix by *λ*, the basic reproductive ratio *R*_0_ is given as follows:

$$R_{0}=\rho_{0}\lambda ,\;\lambda \equiv \rho \left(\left[\begin{array}{ccc} T_{RR} & T_{RH} & 0\\ T_{HR} & T_{HH} & T_{HW}\\ T_{WR} & T_{WH} & T_{WW} \end{array}\right]\right)$$ (8)

The dominant eigenvalue *λ* and the corresponding eigenvectors are obtained numerically by using the power iteration method. As the basic reproductive ratio *R*_0_ is linearly dependent on the epidemiological factor *ρ*_0_, *R*_0_ for various diseases with different infection rates and recovery rates can easily be calculated from Eq (8). Furthermore, we can also calculate the critical value of infection rate *β*_*c*_ for the disease invasion to occur using Eq (8) as *R*_0_ = (*β*_*c*_/*γ*)*λ* = 1.

### Sensitivity analysis of the basic reproductive ratio *R*_0_

The epidemiological influence of each station and each commuting pathway is obtained by applying sensitivity analysis [34,35] to the basic reproductive ratio *R*_0_. Countermeasures such as vaccination/quarantine will decrease the number of susceptible hosts in local populations, leading to a change in the next generation matrix ***L*** → ***L*** + *δ****L***, which then alters the basic reproductive ratio *R*_0_ →*R*_0_ + *δR*_0_. When the change *δ****L*** in the next generation matrix is small, the associated change in the basic reproductive ratio *δR*_0_ can be calculated as follows (see [34,35] for derivation):

$$\delta R_{0}=\frac{v^{t}\delta Lw}{v^{t}w}$$ (9)

where ***ν*** ≡ (***ν***^*R*^, ***ν***^*H*^, ***ν***^*W*^)^*t*^ and ***w*** ≡ (***w***^*R*^, ***w***^*H*^, ***w***^*W*^)^*t*^, where superscript *t* denotes a transpose, with ${\left(v^{m}\right)}^{t}={\left(v_{1}^{m},v_{2}^{m},\cdots ,v_{M}^{m}\right)}^{t}$ and ${\left(w^{n}\right)}^{t}={\left(w_{1}^{m},w_{2}^{m},\cdots ,w_{M}^{m}\right)}^{t}$ for *m*,*n* ∈ {*R*, *H*, *W*} are the left and right eigenvectors associated with the largest eigenvalue *R*_0_ of the next generation matrix (***ν***^*t*^***L*** = *R*_0_***ν***^*t*^, and ***Lw*** = *R*_0_***w***). Each element of the left eigenvector ***ν*** corresponds to the “reproductive value”, which quantifies the effect of initially infected hosts in the associated local population on the exponential phase of the epidemic. In contrast, the elements of the right eigenvector ***w*** correspond to the quasi-stationary distribution of the infected population in the initial exponential phase of the epidemic [34,35]. See S2 Fig and S1 File for the calculated results for the left and right eigenvectors. By using Eq (9), we can calculate the effect of countermeasures against the epidemic such as vaccinations and/or quarantine on the basic reproductive ratio *R*_0_. Here, we have simply assumed that the vaccination will change the susceptible individual to completely immune state and that the quarantine will completely isolate the susceptible individual from the infectious host and prevent the disease transmission. Therefore both vaccination and quarantine are assumed to reduce the number of susceptible individuals.

The effect of decreasing a small number *ϵ* of susceptible individuals from each local population can be calculated as follows. A reduction of *ϵ* susceptible non-commuting individuals from the *k*_0_-th resident population will change the elements of the host population structure matrix as [*δ****T***_*RR*_]_*ik*_ = −*ϵδ*_*ik*_*δ*_*iko*_ and [*δ****T***_*RH*_]_*ik*_ = −*ϵδ*_*ik*_*δ*_*iko*_, where *δ*_*ij*_ is the Kronecker delta defined as *δ*_*ij*_ = 1 if *i* = *j* and *δ*_*ij*_ = 0 if *i* ≠ *j*, leading to a decrease in the basic reproductive ratio *δR*_0_(*k*_0_) of

$$\begin{array}{l} \delta R_{0}^{R}\left(k_{0};\;\epsilon \right)=\frac{\rho_{0}}{v^{t}w}\left[\begin{array}{ccc} v^{R} & v^{H} & v^{W} \end{array}\right]\left[\begin{array}{ccc} \delta T_{RR} & \delta T_{RH} & 0\\ 0 & 0 & 0\\ 0 & 0 & 0 \end{array}\right]\left[\begin{array}{c} w^{R}\\ w^{H}\\ w^{W} \end{array}\right]\;\\ =-\frac{\epsilon \rho_{0}}{v^{t}w}\left(v_{k_{0}}^{R}w_{k_{0}}^{R}+v_{k_{0}}^{R}w_{k_{0}}^{H}\right). \end{array}$$ (10)

Similarly, a decrease of a small number *ϵ* of susceptible commuting individuals who reside in the *i*_0_-th home population and move to the *j*_0_-th work population will change the next generation matrix as ${\left[\delta T_{HR}\right]}_{ik}={\left[\delta T_{HH}\right]}_{ik}=\;-\epsilon \delta_{ik}\delta_{ii_{0}},$ ${\left[\delta T_{HW}\right]}_{ik}=-\epsilon \delta_{ii_{0}}\delta_{kj_{0}},$ ${\left[\delta T_{WR}\right]}_{ik}=-\epsilon \delta_{ij_{0}}\delta_{ki_{0}},$ ${\left[\delta T_{WH}\right]}_{ik}=-\epsilon \delta_{ij_{0}}\delta_{ki_{0}}$, and ${\left[\delta T_{WW}\right]}_{ik}=-\epsilon \delta_{ik}\delta_{kj_{0}},$ leading to a decrease in the basic reproductive ratio of

$$\begin{array}{l} \delta R_{0}^{C}\left(i_{0},j_{0};\epsilon \right)=\;\frac{\rho_{0}}{v^{t}w}\left[\begin{array}{ccc} v^{R} & v^{H} & v^{W} \end{array}\right]\left[\begin{array}{ccc} 0 & 0 & 0\\ \delta T_{HR} & \delta T_{HH} & \delta T_{HW}\\ \delta T_{WR} & \delta T_{WH} & \delta T_{WW} \end{array}\right]\left[\begin{array}{c} w^{R}\\ w^{H}\\ w^{W} \end{array}\right]\;\\ =\;-\frac{\epsilon \rho_{0}}{v^{t}w}(v_{i_{0}}^{H}w_{i_{0}}^{R}+v_{i_{0}}^{H}w_{i_{0}}^{H}+v_{i_{0}}^{H}w_{j_{0}}^{W}+v_{j_{0}}^{W}w_{i_{0}}^{R}+v_{j_{0}}^{W}w_{i_{0}}^{H}+v_{j_{0}}^{W}w_{j_{0}}^{W}). \end{array}$$ (11)

By using Eqs (10) and (11), we can quantify the effect of countermeasures at every station and every commuting pathway by the decrease in the basic reproductive ratio. Because Eq (9) is obtained by first order approximation, it is assumed that *ϵ* is small and, as a result, $\delta R_{0}^{R}$ and $\delta R_{0}^{C}$ are linearly dependent on *ϵ*.

### A centrality measure based on the sensitivity analysis of the basic reproductive ratio *R*_*0*_

Using the results obtained from the sensitivity analysis of the basic reproductive ratio *R*_*0*_ (Eq (9)), we defined a novel centrality measure for each local population (“*R*_*0*_-centrality”) according to its epidemiological influence. When a countermeasure is applied to a single station or single commuting pathway, a larger decrease in the basic reproductive ratio *R*_*0*_ means that the countermeasure is relatively effective and hence the epidemiological influence of that location is high. We therefore defined the *R*_*0*_-centrality as a change in the basic reproductive ratio *R*_*0*_ when a unit number of susceptible individuals are vaccinated/quarantined at each local population. For a non-commuting resident population associated with each station *i* (*i* = 1,2,…,*M*), the *R*_*0*_-centrality is given by

$$C_{i}^{R}\equiv \frac{\delta R_{0}^{R}\left(i;\epsilon \right)}{\epsilon }=-\frac{\partial \rho \left(L\right)}{\partial N_{i}^{R}}=-\rho_{0}\frac{\partial \rho \left(T\right)}{\partial N_{i}^{R}},$$ (12)

where ***T*** = ***L/****ρ*_0_ is the host population structure matrix. The corresponding centrality measure for a commuting population (in other words, for a commuting pathway) for individuals who commute from the *i*-th station to the *j*-th station to work/study, is defined as

$$C_{ij}^{C}\equiv \frac{\delta R_{0}^{C}\left(i,j;\epsilon \right)}{\epsilon }=-\frac{\partial \rho \left(L\right)}{\partial N_{ij}^{C}}=-\rho_{0}\frac{\partial \rho \left(T\right)}{\partial N_{ij}^{C}}.$$ (13)

By calculating the *R*_0_-centrality for every station and every commuting pathway, we are able to quantify the epidemiological influence of all these locations. It should be noted that this centrality measure is linearly dependent on the epidemiological factor *ρ*_0_, enabling it to be applied to a wide range of diseases with different infection rates and recovery rates.

## Results

### *R*_0_-centralities at the major stations in the Tokyo metropolitan area

**Fig 2:**
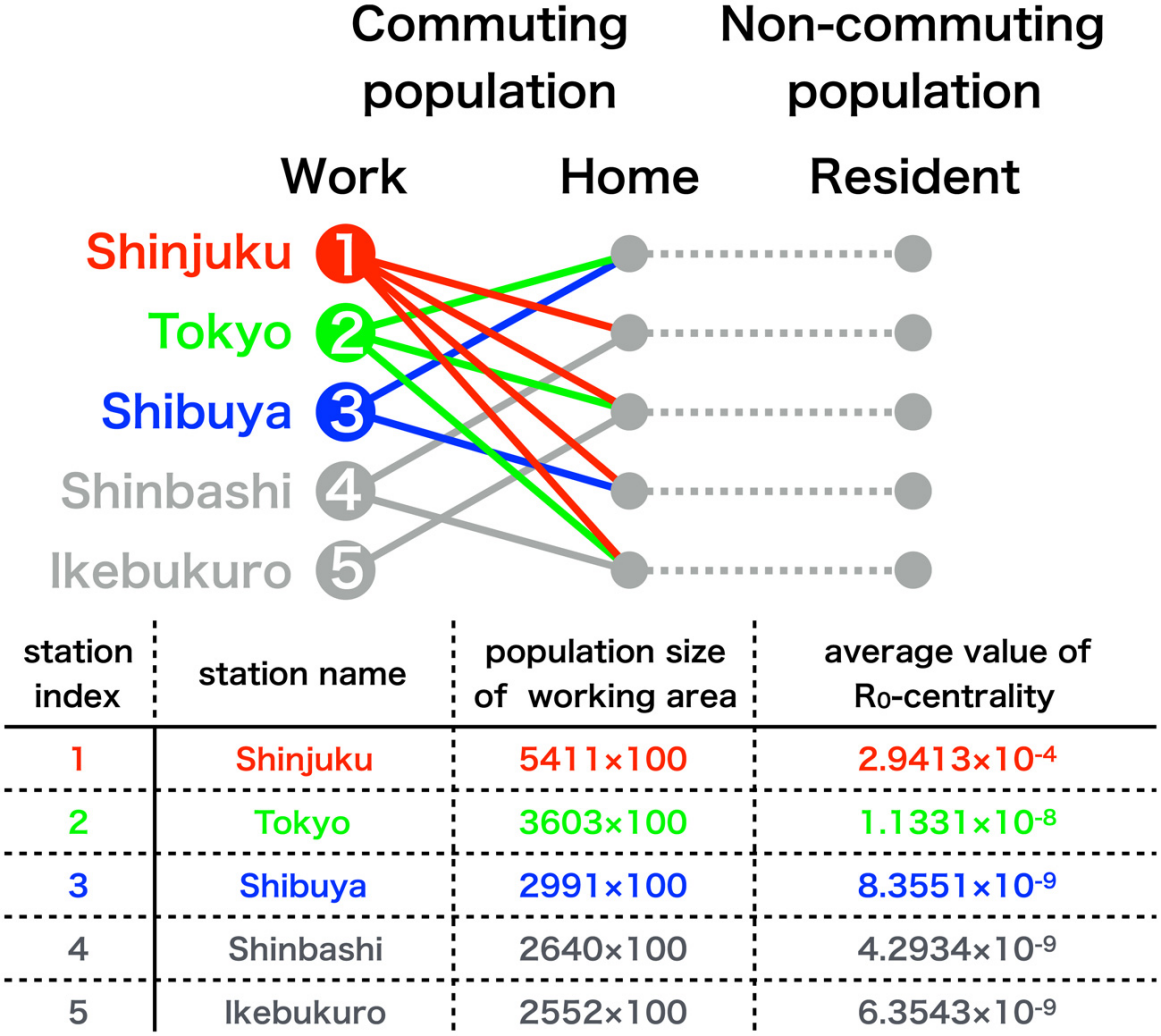
*R*_0_-centralities at the major stations in the Tokyo metropolitan area. The red lines indicate the commuting population who commutes to the Shinjuku station, which is the largest working/studying station in the Tokyo metropolitan area [36]. Similarly, the green lines indicate those for the second largest Tokyo station and the blue lines indicate those for the third largest Shibuya station. The average values of the *R*_0_-centralities of these commuting pathways are given in the top three rows of the table (*R*_0_ = 1.6 is assumed in the calculation).

The epidemiological risk of commuters (commuting population) who are traveling between residing station (home population) and working station (work population) in the Tokyo metropolitan area are evaluated using the *R*_0_-centrality. The populations with the largest *R*_0_-centralities are summarized in Fig 2. We found that among the commuting populations in the Tokyo metropolitan area, those commuting to the largest working population (Shinjuku station) had the largest *R*_0_-centralities. These values are extraordinary higher than any other commuting populations. This can be seen by comparing the mean value of the *R*_0_-centralities averaged over commuting populations travelling to the largest work population (Shinjuku station), to that of the second largest (Tokyo station), the third largest (Shibuya station) and so forth. The population size of the largest work population is only about 1.5 times larger than the second largest work population, in spite of the fact that the *R*_0_-centrality is more than 1,000 times larger. This means that, in preventing the disease invasion, applying countermeasures against an individual in the working population at Shinjuku station is more effective than applying countermeasures against 1,000 individuals in the second largest working population at Tokyo station.

### *R*_0_-centrality for every commuting pathway and residential station in the Tokyo metropolitan area

**Fig 3:**
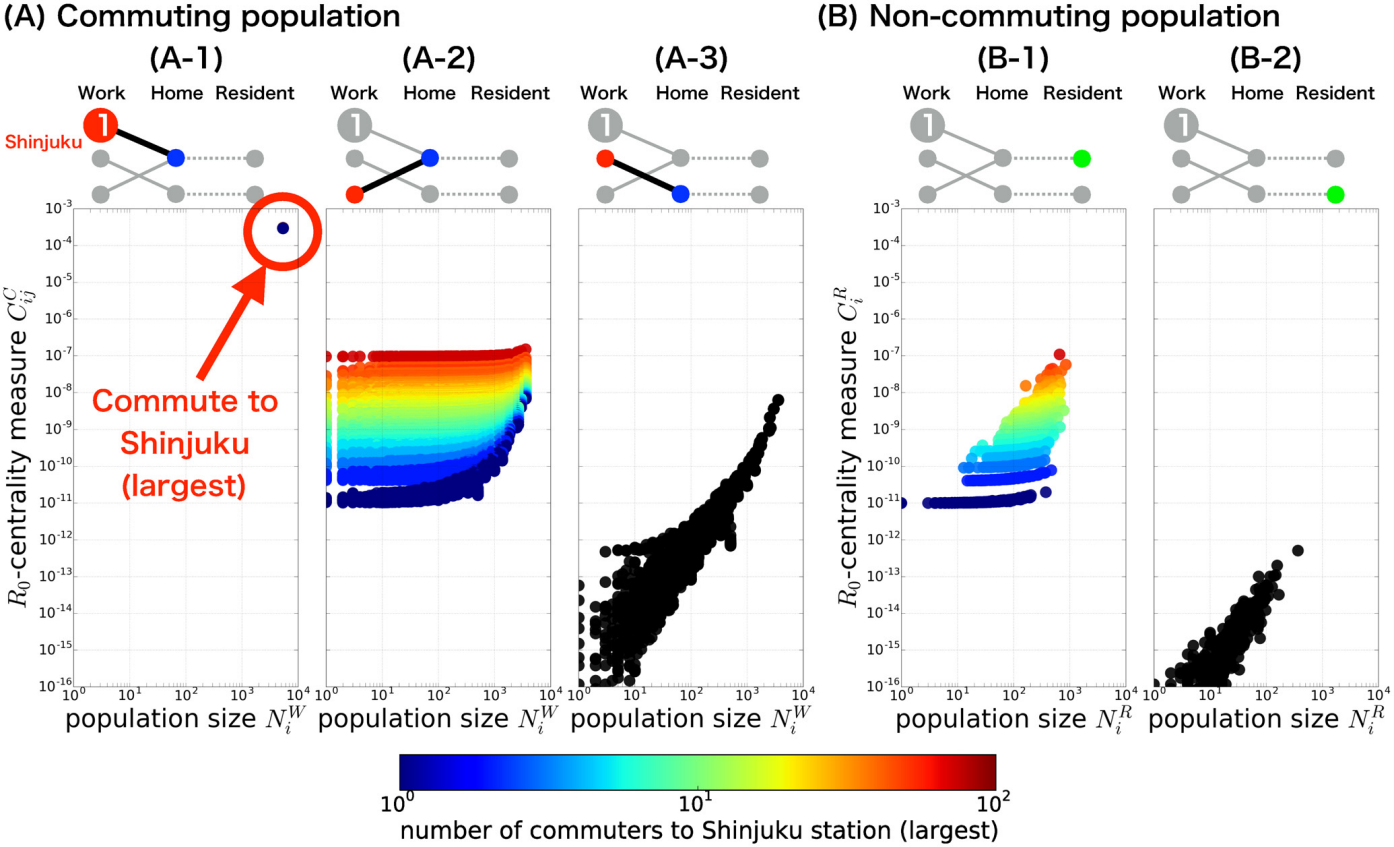
The *R*_0_-centrality for every commuting pathway and residential station in the Tokyo metropolitan area. The *R*_0_-centralities of commuting populations: (A-1), those who directly commute to Shinjuku station; (A-2), those who do not commute to Shinjuku station but share a common resident station with them; (A-3), neither of them, are plotted against the population size of its working population. Similarly, the *R*_0_-centralities of non-commuting population: (B-1), those residing at the station area from which at least one commutes to Shinjuku station; (B-2), those residing at the station area from which no one commutes to Shinjuku station, are plotted against the population size of its resident population.

This distinct influence of Shinjuku station is not limited to those who *directly* commute to Shinjuku station, but extends to wider range of populations through their *indirect* connections (see below) to Shinjuku station. The *R*_0_-centralities of every commuters (commuting population) and residents who do not commute daily (*non-commuting population*) can be summarized by their *closeness* in relationship to the Shinjuku station (Fig 3). Notable distinctions in the *R*_0_-centralities among the classified groups of populations can clearly be explained by their relation to the Shinjuku station. A large difference in the *R*_0_-centralities can been seen between those who directly commute to Shinjuku station and those who do not (compare Fig 3A-1 with other panels in Fig 3). In addition, among those who do not directly commute to the Shinjuku station, but share a common residence station with those who directly travel to Shinjuku (Fig 3A-2 and 3B-1), the *R*_0_-centrality are determined by the number of residents who commute to the Shinjuku station (see the horizontally colored layers). The relation to the Tokyo station (the second largest working population) fails to give any such clear separation (see S3 Fig and compare it with Fig 3). However, when the working population of Shinjuku station is completely removed from the network (e.g., by vaccinating/quarantining all the individuals commuting to Shinjuku station), the Tokyo station (the largest susceptible working population after the removal) has the pivotal role in determining the *R*_0_-centralities as the Shinjuku station did before removal (see S4 Fig). This suggests that the reason why the Shinjuku station has markedly strong influence is just in its largest population size itself, rather than any peculiarity in topological location or connectivity it might have within the network.

### The effect of countermeasures at each of the major stations in the Tokyo metropolitan area

**Fig 4:**
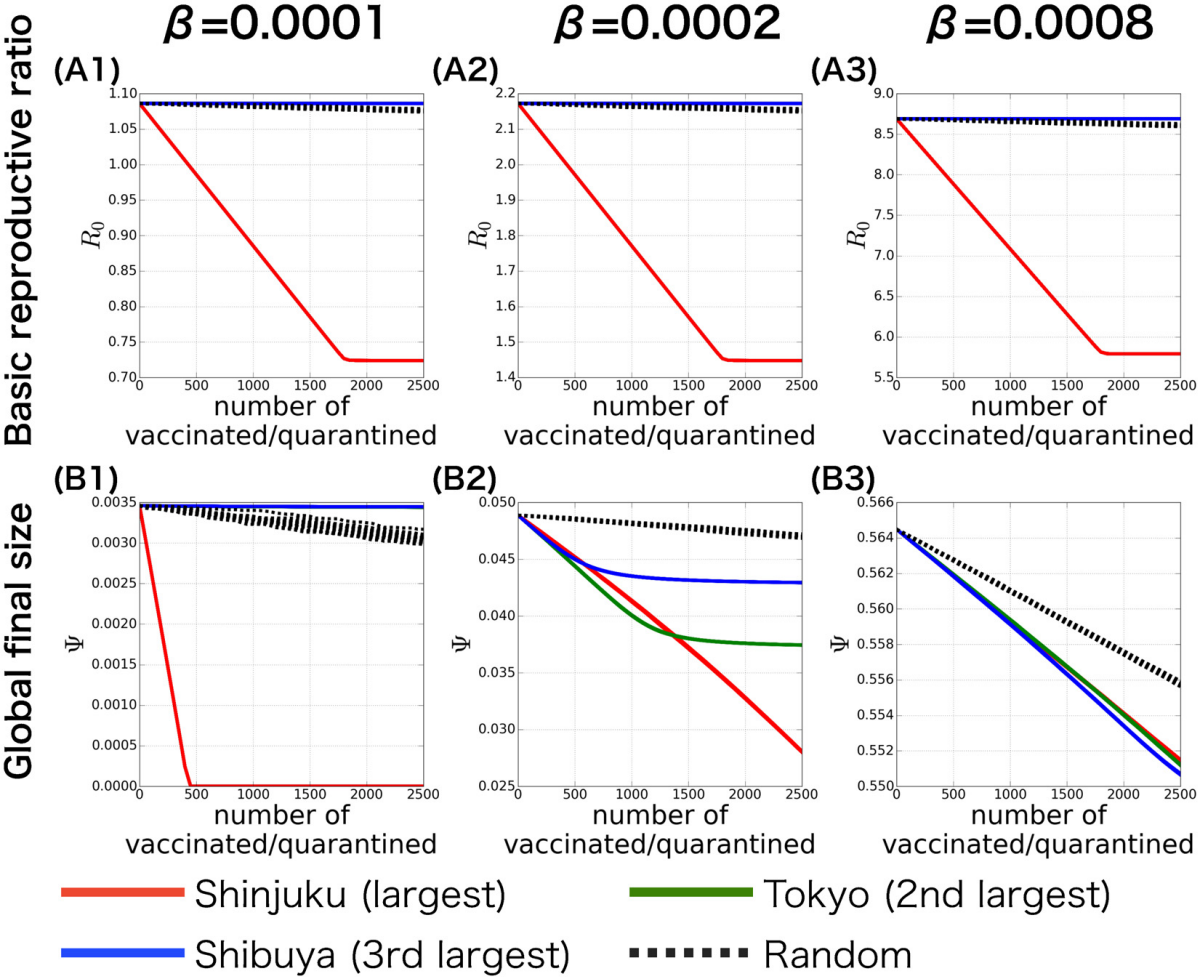
The effect of countermeasures at each of the major stations in the Tokyo metropolitan area. The basic reproductive ratio *R*_0_ (A) and the global final size of epidemic Ψ (B) are given as a function of the number of vaccinated/quarantined, respectively. The vaccination/quarantine is independently applied to each of the following major stations: Shinjuku (the largest working population, red lines), Tokyo (the second largest working population, green lines), and Shibuya (the third largest working population, blue lines). The results of random vaccination/quarantine are given in black dotted lines. Each column denotes the results for different *β*, which is defined as the infection rate from a single infectious host per unit time (relation between *R*_0_ and *β* is given in Methods section).

This remarkable difference between the largest and the second largest working population in terms of their influence on the epidemic dynamics has also been confirmed further. To see this we have calculated the *R*_0_ and the global final size of epidemic Ψ, when the countermeasures are independently applied to the working population of major stations. Here the countermeasures are only applied to a single population while the other populations are kept untouched, this enables us to measure the contribution from the relevant population only. The *R*_0_ is numerically calculated by solving the eigenvalue of the next generation matrix (Eq 7); therefore the results are valid for arbitrary amount of countermeasures. As expected from the results of the *R*_0_-centrality, applying countermeasures to the working area of Shinjuku station effectively decreased *R*_0_; on the other hand, applying countermeasures to the other smaller populations had minimal effect on *R*_0_ (Fig 4A). This dominating effect of Shinjuku station disappears when the number of vaccinated/quarantined exceeds about one thirds of the original population. This can be explained by the fact that at this point the number of susceptible individuals at Shinjuku station becomes smaller than Tokyo station (compare S5 with S6 Figs). Interestingly, the effect of countermeasures on *Ψ* was quite different (Fig 4B). When the infection rate is small (i.e., slightly larger then the disease invasion threshold, S1A Fig) the results were similar to the effect against *R*_0_,—applying countermeasures to the Shinjuku station successfully reduced *Ψ*, while application to other smaller populations has a minimal effect on Ψ (Fig 4B-1). However, for a larger infection rate, applying countermeasures to stations with smaller working population size than Shinjuku station also effectively reduced *Ψ* (Figs 4B-2 and 3). Furthermore, the effect of countermeasure is not always stronger for population with larger population size. This contrasting result in the final size of epidemic can be explained as follows. If infection rate is well above the disease invasion threshold, the final size of epidemic is already nearly fully saturated in the largest populations, and any countermeasures applied there fail to effectively reduce local final size of epidemic in such local populations. In smaller populations where their population sizes are located near the inflation point in the abscissa of the curve of local final size of epidemics (see S1B and S7 Figs), countermeasures can still sensitively reduce the local final size of epidemic. Important lesson drawn from these results are that the optimum intervention can be different when focusing on the prevention of disease invasion (where *R*_0_-centrality is important) than when focusing on the mitigation of the total toll of disease (i.e. reducing *Ψ*) after it had been invaded.

## Discussion

In this study we have introduced a novel centrality measure “*R*_0_-centrality” for each node and edge of metapopulation networks based on sensitivity analysis of the basic reproductive ratio *R*_0_. For the analysis we utilized the actual commute network data of the Tokyo metropolitan area. Using this centrality measure, we found that the local population with the largest population had a marked influence on the epidemic dynamics (i.e., on *R*_0_) compared with the smaller local populations. This result was further confirmed by comparing the final size of the epidemic when quarantine was applied to the largest local population with those applied to the other smaller local populations.

In the theory of networks it is well known that the heterogeneity of a network (e.g., the presence of hubs, i.e., the nodes with a large degree centrality) enhances the severity of an epidemic [15–21] and that a targeted intervention focusing on the hubs becomes important [22–25]. However, previous analyses overlooked what we observed in this study. Only the largest population in a network, among a number of sufficiently large populations, plays an important role in the epidemic. The reason for overlooking this may be due to the centrality measure, degree centrality, they used in their analyses. For the Tokyo metropolitan area, the population size of the largest work population is only approximately 1.5 times the size of the second largest work population, indicating that the ratio of degree centrality is also approximately 1.5; hence, is obviously incapable of explaining the marked difference between the impacts of the largest and second largest work populations in our analysis, which differed by a factor greater than 1,000 (Fig 2). This clearly indicated that the result should stem largely from the dynamical process of the epidemic rather than from the static geometrical structure of the network characterized by the degree centrality of nodes. Moreover, none of the studies based on the other centrality measures of static geometrical structure of networks, such as betweenness centrality [26,27] and k-coreness [28], showed this marked role of the largest population.

The reason why this markedly strong influence of the largest subpopulation exists can be interpreted as follows. For the case where the fraction of commuters between subpopulations is very small, subpopulations are nearly isolated from each other. In such case the basic reproductive ratio of the whole metapopulation is determined solely by that of the largest subpopulation, however small might be the difference between the largest and the second largest. Therefore, applying countermeasures to any other smaller subpopulation has virtually no effect on decreasing the basic reproductive ratio. In terms of *R*_0_-centrality, this corresponds to extremely large *R*_0_-centrality at the largest subpopulation relative to the other’s—the latter tends to zero in the limit of complete isolation. Our result suggests that even though the movements between the subpopulations occur rather extensively in the Tokyo metropolitan area, this prominent effect of the largest subpopulation still exists. We are currently analyzing this effect of the mobility rate on the epidemic dynamics, using the perturbation method (manuscript in preparation), where the next generation matrix is decomposed into an unperturbed matrix, which describes the local infection within each isolated subpopulation, and a perturbed matrix, which describes the spread of infection between subpopulations. The magnitude of this perturbation can be given as a ratio $\eta =\underset{ij}{\text{max}}\left(N_{ij}^{C}\right)/\underset{i}{\text{max}}\left(N_{i}^{W}\right)\approx 100/5000$ in our model of the Tokyo metropolitan area. According to the analytical form of the *R*_0_-centrality derived by perturbation with respect to *η*, we found that the *R*_0_-centrality of the largest subpopulation should be factor 1/*η*^2^ = 2500 times larger than that of the second largest. We believe that this explains the reason why the largest population has a markedly strong influence on the preventing the invasion of the disease.

The *R*_0_-centrality that we introduced includes information from not just the static geometric structure of the network but also from dynamic aspects of the epidemic process, and we believe that because of this reason it is capable of revealing the markedly strong influence of the largest node. It should be noted that the centrality measure introduced by Klemm et al. (the “dynamic influence”) [29] has a similar perspective to our centrality measure, with both taking into account the dynamical aspects of the epidemic process. They calculated the leading left eigenvector of the characteristic matrix, obtained from the linearized system of ordinary differential equations that describe the network epidemic, and defined the centrality of each node as the corresponding element of the leading left eigenvector. In contrast, our *R*_0_-centrality was defined as the sensitivity of each node to *R*_0_, which depended on the dominant eigenvalue and the leading right and left eigenvectors of the next generation matrix. Despite this difference, both measures quantified how much each node (i.e., local population) contributed to the rate of initial exponential growth of the epidemic in the total population. However, Klemm et al.’s results did not show a distinct separation of the impacts of nodes into groups, as observed in our results (S2 Fig). This may be because of the difference in the network structure; they used an individual contact network whereas we used a metapopulation network.

Compared with the previous centrality measures, our proposed centrality measure has several practical advantages. In epidemics on a network, it is well established that the hubs are the vital part of the network and are the major targets for quarantine. Our results show that slight differences in the population size of local populations are greatly amplified in their impact on the epidemic dynamics, particularly between the largest population and the other smaller populations. This also indicates that, when applying quarantine to a metapopulation network, it is important to target the local population of the largest number of non-quarantined individuals, not just fixing the target to the largest population before the quarantine is started. Another practical implication is that, as the basic reproductive ratio *R*_0_ itself is the control target; we were able to evaluate the minimum countermeasures necessary for prevention of disease invasion. Reducing the basic reproductive ratio below the threshold value (*R*_0_ < 1) is sufficient for disease prevention. Finally, as our *R*_0_-centrality of a node is defined directly in terms of its impact on epidemic dynamics (e.g., the removal of susceptible individuals from a node with a *R*_0_-centrality that is double in size will have double impact on the basic reproductive ratio), we can estimate the effect of an intervention strategy much more precisely than when using the other centrality measures. For example, suppose we have to choose local populations to apply countermeasures based both on the epidemiological impact and social and economic impact, we can adjust the strategies to minimize social/economic damage while keeping the required reduction in epidemic impact (*R*_0_-centrality).

In order to apply our result in practical intervention strategies against various infectious diseases, several factors should be included. First, infection in the commuter train during the commute is not included in our model. The inclusion of this effect will drastically increase the complexity of the epidemic process and its theoretical analysis. Therefore, the application of our method (i.e., centrality measure based on *R*_0_ sensitivity analysis) to airborne diseases such as influenza, tuberculosis, and measles might require inclusion of this effect [45,46] and could lead to different outcome. However, Cooley et al. have shown, by an agent-based simulation of influenza epidemic using the mobility data of the New York City, that the transmissions occurring on the subway are 4~5% of the total infections [47], indicating that the infections in commuter trains could be relatively small. Second, host structures in a local population such as age groups, community groups, schools, offices, and factories are not considered and assumed as a well mixed population in this study. Instead, we focused on modeling the heterogeneity in local population connectivity in the commute network, but the intra-subpopulation structure would be another important refinement to our analysis (for example, see Mossong et al. [48] for social contact patterns). However, extension of our *R*_0_-centrality to include these factors can be performed in the same framework that we have described in this study. Lastly, the targeted intervention should be applied by recalculating the *R*_0_-centrality along with the quarantine procedure, since the quarantine itself will change the centrality measure. This update will gave a more accurate estimate of the epidemiological impact and improves the effectiveness of the targeted intervention strategy.

## Conclusion

Previous analyses overlooked what we observed in this study: only the largest population in a network plays an extraordinary important role in the epidemic. The reason for overlooking this may be due to different centrality measure, degree centrality, used in the previous studies. For the Tokyo metropolitan area, the population size of the largest work population is only approximately 1.5 times the size of the second largest work population, indicating that the ratio of degree centrality is also approximately 1.5; hence, it is obviously incapable of explaining the marked difference between the impacts of the largest and second largest work populations in our analysis, which differed by a factor greater than 1,000. None of the studies based on other centrality measures showed this marked role of the largest population. By using the *R*_0_-centrality, we have revealed that a slight difference in the population size are greatly amplified in their impact on the epidemic dynamics, particularly between the largest population and the other smaller populations.

## Supporting Information

S1 FigThe final size of epidemic for infectious disease spread in the Tokyo metropolitan area.(A) The global final size *Ψ* of the epidemic for *r* = 0 (blue line) and *r* = 1 (red lines) are plotted against the infection rate *β* (*r*: ratio of non-commuting individuals to commuting individuals, see Methods section for details). For *r* = 0, where all the population would commute, the result for the commuting population *Ψ*^*C*^ (in this case the same as the result for the total population) is only present as a blue solid line. For *r* = 1, the type of line indicates the result for the commuting population *Ψ*^*C*^ (red dotted line), non-commuting population *Ψ*^*R*^ (red dashed line), and total population *Ψ* (red solid line), respectively. Note that the result for the total population with *r* = 1 (red solid line) overlaps with the result for commuting population with *r* = 0 (blue solid lines) and is not visible on the figure. As the infection rate *β* exceeds a threshold value, the global final size Ψ of the epidemic becomes non-zero and increases along with the infection rate, for both values of *r*. According to the analysis of the basic reproductive ratio, the threshold value of infection *β*_*c*_ is given as *β*_*c*_ = 9.210485 × 10^−5^ for *r* = 0 and *β*_*c*_ = 9.207523 × 10^−5^ for *r* = 1. These values are in good agreement with the results obtained for the final size of the epidemic. (B) The local final size of the epidemic at work population ($\Psi_{i}^{W}$), home population ($\Psi_{i}^{H}$), and resident population ($\Psi_{i}^{R}$) of each station are plotted against its local population size in Figs B1, B2, and B3, respectively. The results for different infection rates are denoted by different colors, here *r* = 1 is used for the calculation. There is a sigmoidal dependence of local final size of epidemic on its population size, such that the local final size is small when the population size is small and as the population size becomes larger it will increase until it saturates to one at the larger limit. Here the location of the steep transition point will shift to the smaller side as the infection rate becomes larger. This point will become relevant in relation to the effect of countermeasures on the final size of epidemic (see Fig 4B).(PDF)

S2 FigThe left and right eigenvectors corresponding to the dominant eigenvalue of next generation matrix for infectious disease spread in the Tokyo metropolitan area.(A) The element of the left eigenvector ($v_{i}^{W}$, $v_{i}^{H}$,$v_{i}^{R}$) that gives the reproductive value of infection at each local population is plotted against its local population size ($N_{i}^{W}$, $N_{i}^{H}$,$N_{i}^{R}$), where each dot represents a single station. The “dynamic influence” introduced by Klemm et al. [29] corresponds to this value, except that they have calculated the eigenvector of the Jacobian matrix and not the next generation matrix. (B) The element of right eigenvector ($w_{i}^{W}$, $w_{i}^{H}$,$w_{i}^{R}$) that gives the relative fraction of infected individuals at each local population in an exponentially growing phase is plotted against its local population size ($N_{i}^{W}$,$N_{i}^{H}$,$N_{i}^{R}$), where each dot represents a single station. Results for the commuting population at each work population and home population are given in (A1, B1) and (A2, B2), respectively and the results for the non-commuting resident population at each station are given in (A3, B3). The color of each dot shows their relationship with the largest work population (Shinjuku station). Black diamonds marked with a red circle in (A1) and (B1) correspond to the largest work population, and other work populations are represented by black dots. Each colored dot in (A2, 3) and (B2, 3) corresponds to a station that has at least one commuter that travels to the largest work population and the color indicates the number of commuters who go there. Black dots correspond to stations with no commuters to the largest work population. For both commuting and non-commuting populations, the elements of the leading left and right eigenvectors were separated into two distinct groups, which can be interpreted from their relationship with the largest work population. The strong dependence of the *R*_**0**_-centrality on the Shinjuku station originates from this characteristic.(PDF)

S3 FigThe *R*_0_-centrality for every commuting pathway and residential station in the Tokyo metropolitan area (the same data as Fig 2 presented in relation to the second largest work population).The *R*_**0**_-centrality for each commuting population (each dot in Fig A corresponds to a single commuting pathway) and non-commuting population (each dot in Fig B corresponds to a single residential station) are given in accordance with the relation to the working population at Tokyo station. The schematic illustration above each panel describes its relationship. The *R*_**0**_-centralities in the commuting populations (A-1), those who commute directly to Tokyo station, (A-2), those who do not commute to Tokyo station but share a common resident station with them, (A-3): neither of them, are plotted against the population size of its working population ($N_{j}^{W}$). Similarly, the *R*_**0**_-centralities of non-commuting population (B-1), those residing at the station area from which at least one commutes to Tokyo station, and (B-2), those residing at the station area from which no one commutes to Tokyo station, are plotted against the population size of its resident population ($N_{j}^{R}$). The color of dots indicates the number of commuters to the working population at Tokyo station.(PDF)

S4 Fig The *R*_0_-centrality for every commuting pathway and residential station in the Tokyo metropolitan area, after vaccinating/quarantining every individual from the largest working population at Shinjuku station. The *R*_**0**_-centrality for each commuting population and non-commuting population after the vaccinating/quarantining all the individual from the largest working population at Shinjuku station, are given in accordance with the relation to the working population at Tokyo station (currently the largest susceptible work population after the removal of Shinjuku station) and Shibuya station (currently the second largest susceptible work population) are given in (A for commuting population, B for non-commuting population) and (C for commuting population, D for non-commuting population), respectively. The schematic illustration above each panel describes its relationship. The color of dots indicates the number of susceptible commuters to the working population at Tokyo station in (A, B) and to the working population at Shibuya station in (C, D).
(PDF)

S5 Fig The *R*_0_-centrality for every commuting pathway and residential station in the Tokyo metropolitan area, after vaccinating/quarantining 1,700 individuals from the largest working population at Shinjuku station. The *R*_**0**_-centrality for each commuting population and non-commuting population after vaccinating/quarantining 1,700 individuals from the largest working population at Shinjuku station, are given in accordance with the relation to the working population at Shinjuku station (the largest susceptible work population) and Tokyo station (the second largest susceptible work population) are given in (A for commuting population, B for non-commuting population) and (C for commuting population, D for non-commuting population), respectively. The schematic illustration above each panel describes its relationship. The color of dots indicates the number of susceptible commuters to the working population at Shinjuku station in (A, B) and to the working population at Tokyo station in (C, D).
(PDF)

S6 Fig The *R*_0_-centrality for every commuting pathway and residential station in the Tokyo metropolitan area, after vaccinating/quarantining 1,900 individuals from the largest working population at Shinjuku station. The *R*_**0**_-centrality for each commuting population and non-commuting population after vaccinating/quarantining 1,900 individuals from the largest working population at Shinjuku station, are given in accordance with the relation to the working population at Shinjuku station (currently the second largest susceptible work population after vaccination) and Tokyo station (currently the largest susceptible work population after vaccination) are given in (A for commuting population, B for non-commuting population) and (C for commuting population, D for non-commuting population), respectively. The schematic illustration above each panel describes its relationship. The color of dots indicates the number of susceptible commuters to the working population at Shinjuku station in (A, B) and to the working population at Tokyo station in (C, D).
(PDF)

S7 Fig The effect of countermeasures on the local final size of epidemic at each major station in the Tokyo metropolitan area. The change in the local final size of epidemic when the vaccination/quarantine is independently applied to the working population of each major station, Shinjuku, Tokyo, and Shibuya are given in (A), (B), and (C), respectively. Here, the vaccination/quarantine is applied to the relevant population only and the other populations are kept untouched. The result for vaccinating 0 (red circle dot), 1,000 (green circle dot) and 2,000 (blue circle dot) individuals are given and the local final sizes of epidemic at each work population are plotted against its local population size. Each panel corresponds to results for different infection rate. The sigmoidal profiles observed in S1B Fig are also evident here; for larger infection rate the transition point will shift to the smaller side. The overall shapes are not altered by the vaccination/quarantine, except for the relevant vaccinated/quarantined population. This is because the number of vaccinated/quarantined is minimal compare to the total population size (i.e., less than 1%), so the effect of vaccination/quarantine is limited to the particular population only. A black arrow denotes the decrease of local final size of epidemic at each vaccinated/quarantined population.
(PDF)

S1 File Derivation of the next generation matrix *L*. The derivation of the next generation matrix *L* for an infectious disease in a metropolitan area is given.
(DOCX)

S2 File Final size of epidemic. The definition of the final size of epidemic and the derivation of the final size equation are given.
(DOCX)
