# Supplementary figures and images for: Spotting Epidemic Keystones by R0 Sensitivity Analysis: High-Risk Stations in the Tokyo Metropolitan Area
Source: PLoS One. 2016 Sep 8;11(9):e0162406. doi: 10.1371/journal.pone.0162406 (PMC5015857; doi:10.1371/journal.pone.0162406)

**(A)** Global final size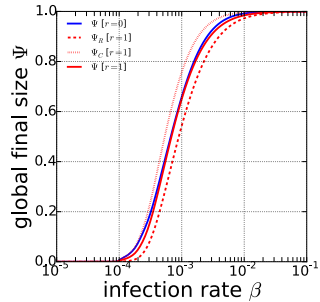**(B1)** Local final size (Work)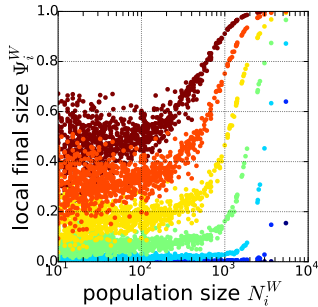**(B2)** Local final size (Home)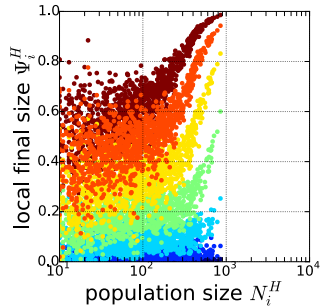**(B3)** Local final size (Resident)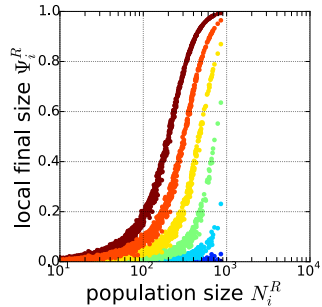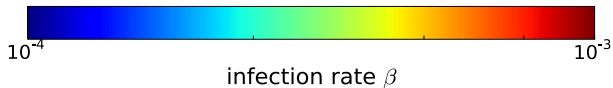

Supplement: S1 Fig — (A) The global final size Ψ of the epidemic for r = 0 (blue line) and r = 1 (red lines) are plotted against the infection rate β (r: ratio of non-commuting individuals to commuting individuals, see Methods section for details). For r = 0, where all the population would commute, the result for the commuting population ΨC (in this case the same as the result for the total population) is only present as a blue solid line. For r = 1, the type of line indicates the result for the commuting population ΨC (red dotted line), non-commuting population ΨR (red dashed line), and total population Ψ (red solid line), respectively. Note that the result for the total population with r = 1 (red solid line) overlaps with the result for commuting population with r = 0 (blue solid lines) and is not visible on the figure. As the infection rate β exceeds a threshold value, the global final size Ψ of the epidemic becomes non-zero and increases along with the infection rate, for both values of r. According to the analysis of the basic reproductive ratio, the threshold value of infection βc is given as βc = 9.210485 × 10−5 for r = 0 and βc = 9.207523 × 10−5 for r = 1. These values are in good agreement with the results obtained for the final size of the epidemic. (B) The local final size of the epidemic at work population (ΨiW), home population (ΨiH), and resident population (ΨiR) of each station are plotted against its local population size in Figs B1, B2, and B3, respectively. The results for different infection rates are denoted by different colors, here r = 1 is used for the calculation. There is a sigmoidal dependence of local final size of epidemic on its population size, such that the local final size is small when the population size is small and as the population size becomes larger it will increase until it saturates to one at the larger limit. Here the location of the steep transition point will shift to the smaller side as the infection rate becomes larger. This point will becom [file pone.0162406.s001.pdf]

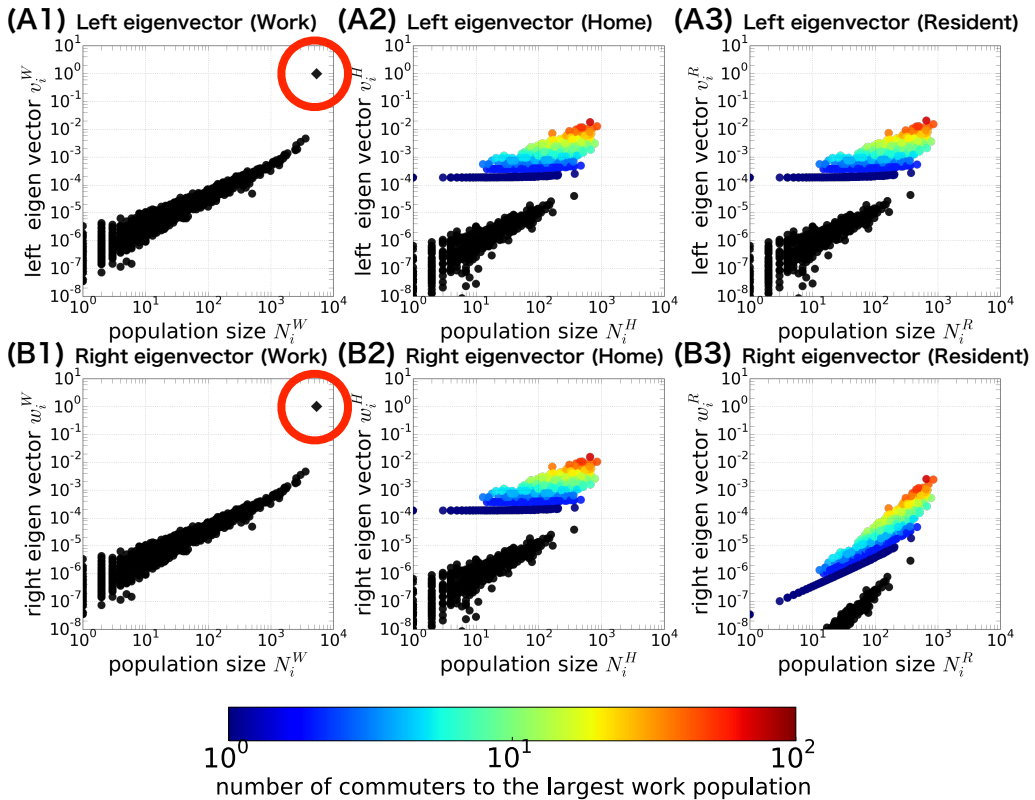

Supplement: S2 Fig — (A) The element of the left eigenvector (viW, viH,viR) that gives the reproductive value of infection at each local population is plotted against its local population size (NiW, NiH,NiR), where each dot represents a single station. The “dynamic influence” introduced by Klemm et al. [29] corresponds to this value, except that they have calculated the eigenvector of the Jacobian matrix and not the next generation matrix. (B) The element of right eigenvector (wiW, wiH,wiR) that gives the relative fraction of infected individuals at each local population in an exponentially growing phase is plotted against its local population size (NiW,NiH,NiR), where each dot represents a single station. Results for the commuting population at each work population and home population are given in (A1, B1) and (A2, B2), respectively and the results for the non-commuting resident population at each station are given in (A3, B3). The color of each dot shows their relationship with the largest work population (Shinjuku station). Black diamonds marked with a red circle in (A1) and (B1) correspond to the largest work population, and other work populations are represented by black dots. Each colored dot in (A2, 3) and (B2, 3) corresponds to a station that has at least one commuter that travels to the largest work population and the color indicates the number of commuters who go there. Black dots correspond to stations with no commuters to the largest work population. For both commuting and non-commuting populations, the elements of the leading left and right eigenvectors were separated into two distinct groups, which can be interpreted from their relationship with the largest work population. The strong dependence of the R0-centrality on the Shinjuku station originates from this characteristic. (PDF) [file pone.0162406.s002.pdf]

# (A) Commuting population

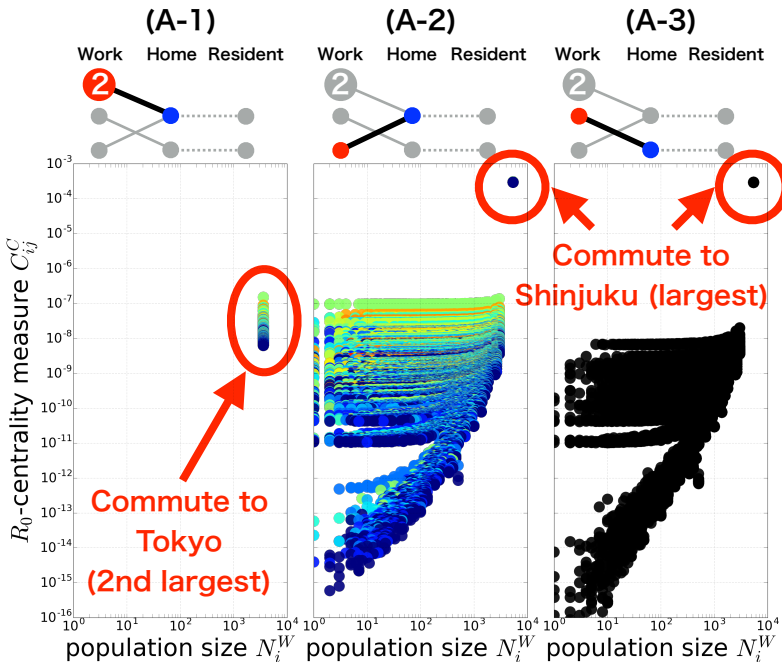

# (B) Non-commuting population

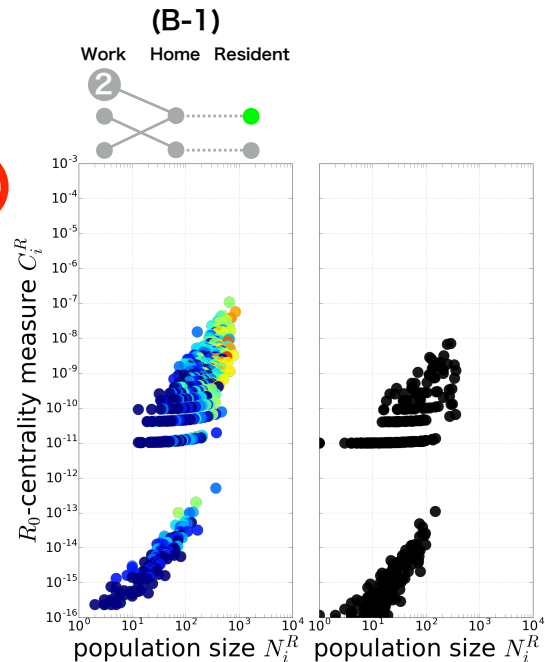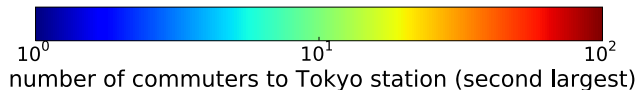

Supplement: S3 Fig — The R0-centrality for each commuting population (each dot in Fig A corresponds to a single commuting pathway) and non-commuting population (each dot in Fig B corresponds to a single residential station) are given in accordance with the relation to the working population at Tokyo station. The schematic illustration above each panel describes its relationship. The R0-centralities in the commuting populations (A-1), those who commute directly to Tokyo station, (A-2), those who do not commute to Tokyo station but share a common resident station with them, (A-3): neither of them, are plotted against the population size of its working population (NjW). Similarly, the R0-centralities of non-commuting population (B-1), those residing at the station area from which at least one commutes to Tokyo station, and (B-2), those residing at the station area from which no one commutes to Tokyo station, are plotted against the population size of its resident population (NjR). The color of dots indicates the number of commuters to the working population at Tokyo station. (PDF) [file pone.0162406.s003.pdf]

Shinjuku

Tokyo

Shibuya

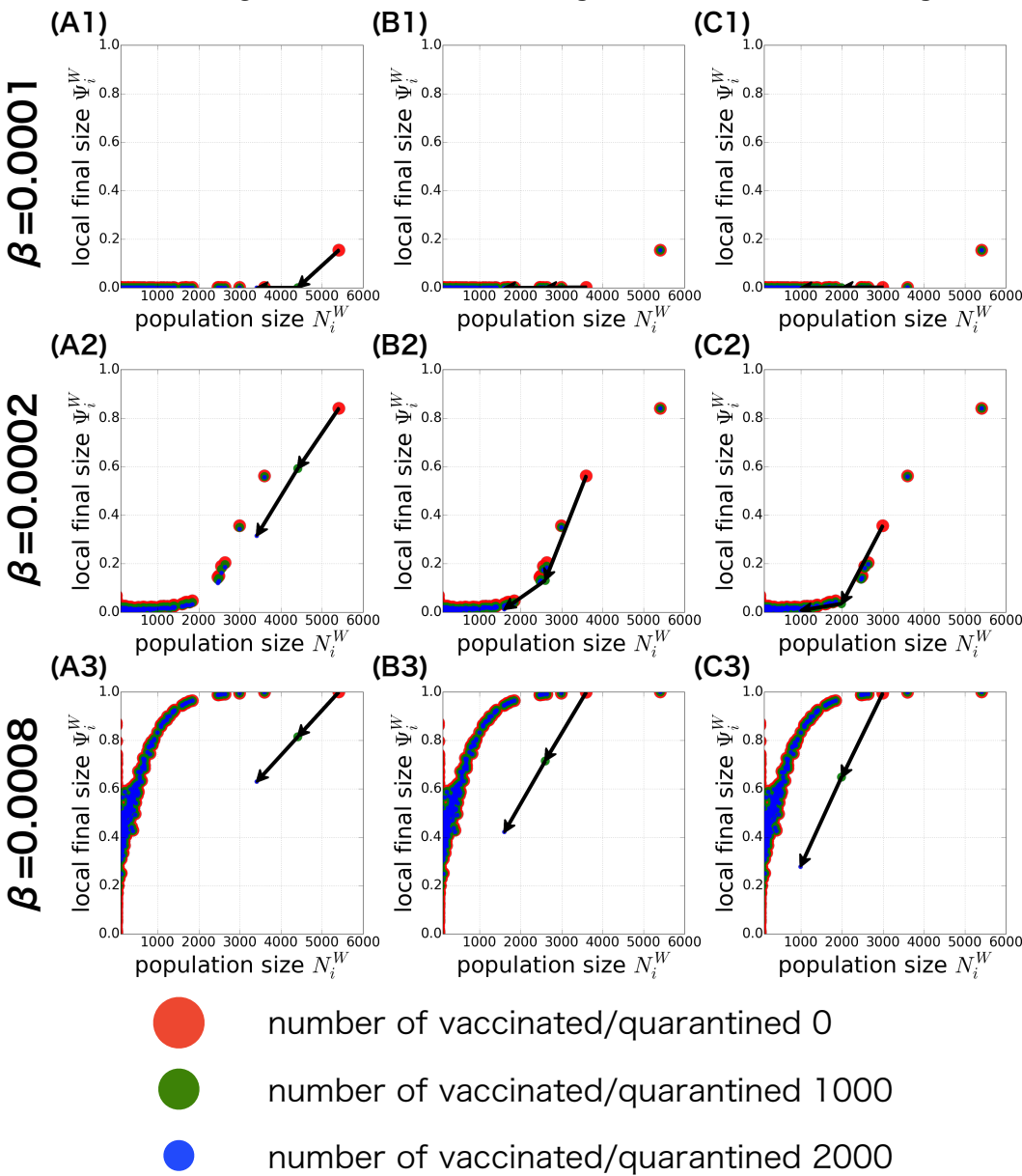

Supplement: S7 Fig — The change in the local final size of epidemic when the vaccination/quarantine is independently applied to the working population of each major station, Shinjuku, Tokyo, and Shibuya are given in (A), (B), and (C), respectively. Here, the vaccination/quarantine is applied to the relevant population only and the other populations are kept untouched. The result for vaccinating 0 (red circle dot), 1,000 (green circle dot) and 2,000 (blue circle dot) individuals are given and the local final sizes of epidemic at each work population are plotted against its local population size. Each panel corresponds to results for different infection rate. The sigmoidal profiles observed in S1B Fig are also evident here; for larger infection rate the transition point will shift to the smaller side. The overall shapes are not altered by the vaccination/quarantine, except for the relevant vaccinated/quarantined population. This is because the number of vaccinated/quarantined is minimal compare to the total population size (i.e., less than 1%), so the effect of vaccination/quarantine is limited to the particular population only. A black arrow denotes the decrease of local final size of epidemic at each vaccinated/quarantined population. (PDF) [file pone.0162406.s007.pdf]
