# Supplementary material for: Spotting Epidemic Keystones by R0 Sensitivity Analysis: High-Risk Stations in the Tokyo Metropolitan Area
Source: PLoS One. 2016 Sep 8;11(9):e0162406. doi: 10.1371/journal.pone.0162406 (PMC5015857; doi:10.1371/journal.pone.0162406)
Supplement: S4 Fig — The R0-centrality for each commuting population and non-commuting population after the vaccinating/quarantining all the individual from the largest working population at Shinjuku station, are given in accordance with the relation to the working population at Tokyo station (currently the largest susceptible work population after the removal of Shinjuku station) and Shibuya station (currently the second largest susceptible work population) are given in (A for commuting population, B for non-commuting population) and (C for commuting population, D for non-commuting population), respectively. The schematic illustration above each panel describes its relationship. The color of dots indicates the number of susceptible commuters to the working population at Tokyo station in (A, B) and to the working population at Shibuya station in (C, D). (PDF) [file pone.0162406.s004.pdf]

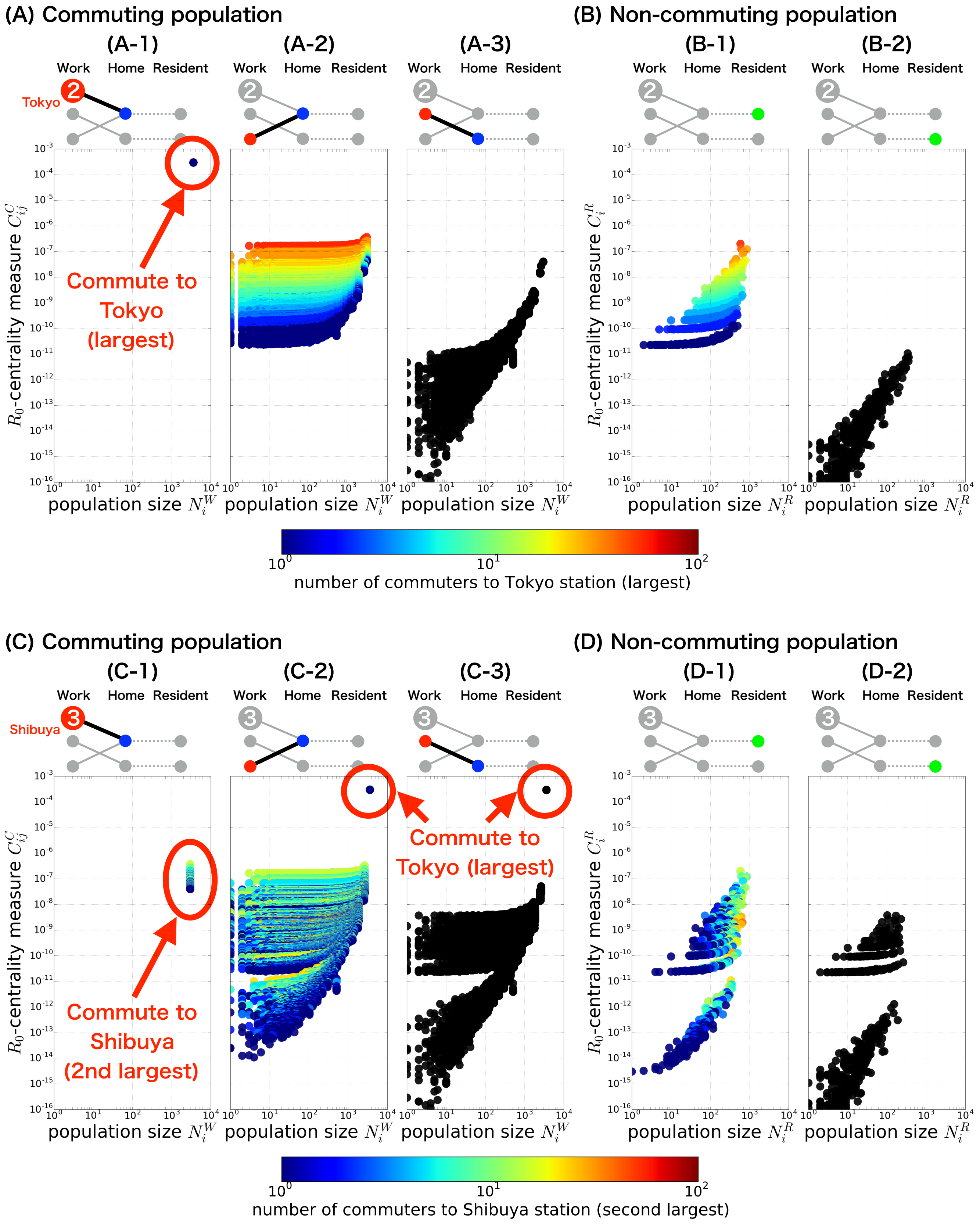

(A) Commuting population
(B) Non-commuting population
(A-1)
(A-2)
(A-3)
(B-1)
(B-2)
Work Home Resident
Tokyo
Commute to Tokyo (largest)
R_0-centrality measure C_{ij}^C
R_0-centrality measure C_i^R
population size N_i^W
population size N_i^R
number of commuters to Tokyo station (largest)
(C) Commuting population
(D) Non-commuting population
(C-1)
(C-2)
(C-3)
(D-1)
(D-2)
Shibuya
Commute to Shibuya (2nd largest)
Commute to Tokyo (largest)
number of commuters to Shibuya station (second largest)
