# Supplementary material for: Spotting Epidemic Keystones by R0 Sensitivity Analysis: High-Risk Stations in the Tokyo Metropolitan Area
Source: PLoS One. 2016 Sep 8;11(9):e0162406. doi: 10.1371/journal.pone.0162406 (PMC5015857; doi:10.1371/journal.pone.0162406)
Supplement: S5 Fig — The R0-centrality for each commuting population and non-commuting population after vaccinating/quarantining 1,700 individuals from the largest working population at Shinjuku station, are given in accordance with the relation to the working population at Shinjuku station (the largest susceptible work population) and Tokyo station (the second largest susceptible work population) are given in (A for commuting population, B for non-commuting population) and (C for commuting population, D for non-commuting population), respectively. The schematic illustration above each panel describes its relationship. The color of dots indicates the number of susceptible commuters to the working population at Shinjuku station in (A, B) and to the working population at Tokyo station in (C, D). (PDF) [file pone.0162406.s005.pdf]

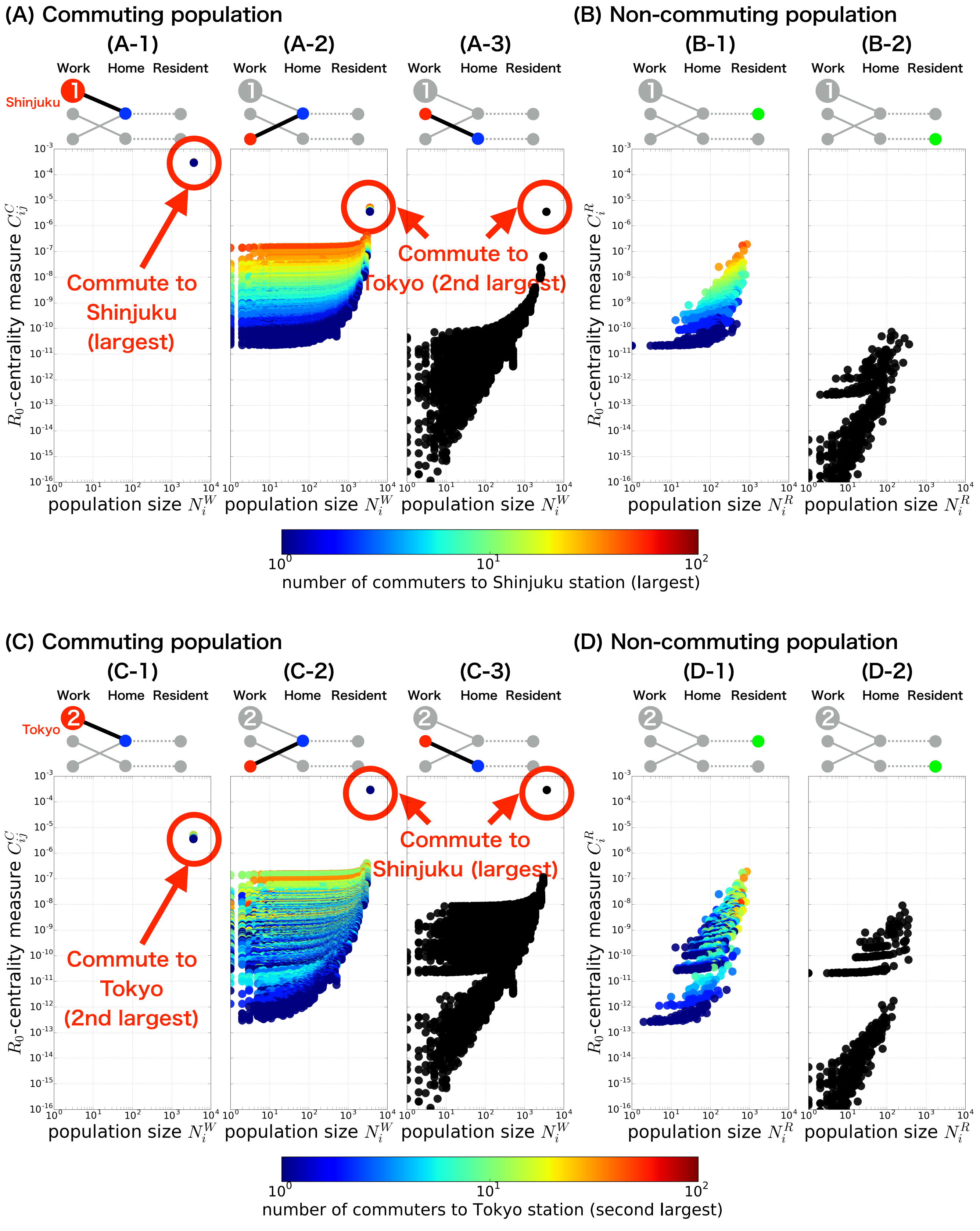

(A) Commuting population
(A-1)
(A-2)
(A-3)
(B) Non-commuting population
(B-1)
(B-2)
Work
Home
Resident
Shinjuku
Commute to Shinjuku (largest)
Commute to Tokyo (2nd largest)
R_0-centrality measure C^C_{ij}
R_0-centrality measure C^R_i
population size N^W_i
population size N^R_i
number of commuters to Shinjuku station (largest)
(C) Commuting population
(C-1)
(C-2)
(C-3)
(D) Non-commuting population
(D-1)
(D-2)
Tokyo
Commute to Tokyo (2nd largest)
Commute to Shinjuku (largest)
number of commuters to Tokyo station (second largest)
