# Supplementary material for: Spotting Epidemic Keystones by R0 Sensitivity Analysis: High-Risk Stations in the Tokyo Metropolitan Area
Source: PLoS One. 2016 Sep 8;11(9):e0162406. doi: 10.1371/journal.pone.0162406 (PMC5015857; doi:10.1371/journal.pone.0162406)
Supplement: S6 Fig — The R0-centrality for each commuting population and non-commuting population after vaccinating/quarantining 1,900 individuals from the largest working population at Shinjuku station, are given in accordance with the relation to the working population at Shinjuku station (currently the second largest susceptible work population after vaccination) and Tokyo station (currently the largest susceptible work population after vaccination) are given in (A for commuting population, B for non-commuting population) and (C for commuting population, D for non-commuting population), respectively. The schematic illustration above each panel describes its relationship. The color of dots indicates the number of susceptible commuters to the working population at Shinjuku station in (A, B) and to the working population at Tokyo station in (C, D). (PDF) [file pone.0162406.s006.pdf]

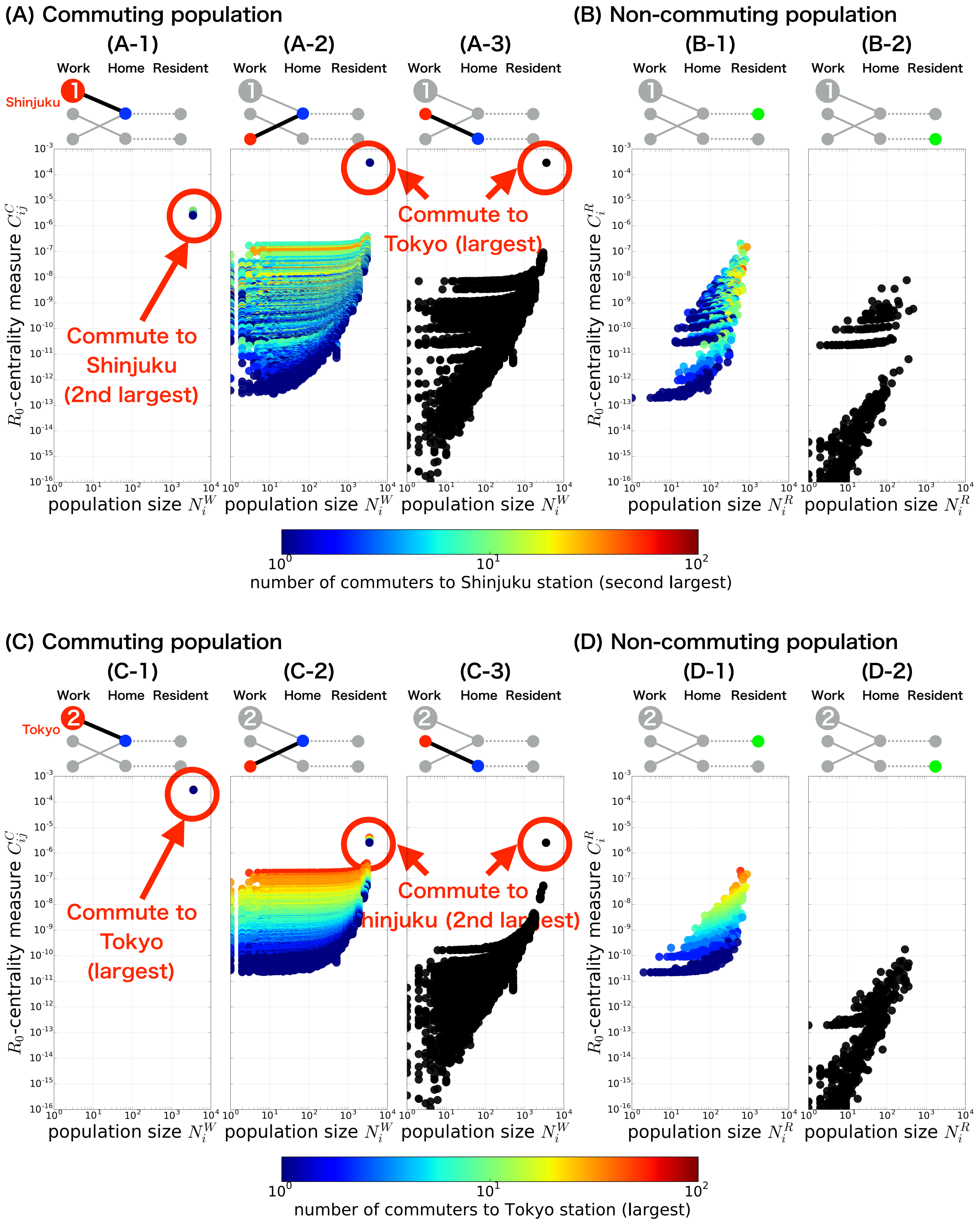
(A) Commuting population
(B) Non-commuting population
(A-1)
(A-2)
(A-3)
(B-1)
(B-2)
Work
Home
Resident
Shinjuku
Commute to Shinjuku (2nd largest)
Commute to Tokyo (largest)
R_0-centrality measure C_{ij}^C
R_0-centrality measure C_i^R
population size N_i^W
population size N_i^R
number of commuters to Shinjuku station (second largest)
(C) Commuting population
(D) Non-commuting population
(C-1)
(C-2)
(C-3)
(D-1)
(D-2)
Tokyo
Commute to Tokyo (largest)
Commute to Shinjuku (2nd largest)
number of commuters to Tokyo station (largest)
