# Supplementary material for: Spotting Epidemic Keystones by R0 Sensitivity Analysis: High-Risk Stations in the Tokyo Metropolitan Area
Source: PLoS One. 2016 Sep 8;11(9):e0162406. doi: 10.1371/journal.pone.0162406 (PMC5015857; doi:10.1371/journal.pone.0162406)
Supplement: S1 File — The derivation of the next generation matrix L for an infectious disease in a metropolitan area is given. (DOCX) [file pone.0162406.s008.docx]

**S1 File. Derivation of the next generation matrix** $\boldsymbol{L}$

Derivation of the next generation matrix $\boldsymbol{L}$ for an infectious disease in a metropolitan area is given. By applying a linear approximation $S_{i}^{R}\left( t \right)\cong N_{i}^{R}$ and $S_{ij}^{C}\left( t \right)\cong N_{ij}^{C}$ to the integral forms of Eqs. (2), (5), we have the following renewal equations.

|  | $I_{i}^{R}\left( t \right)=\beta\int_{o}^{t} e^{-\gamma\tau}\left[ 2I_{i}^{R}\left( t-\tau\right)+I_{i}^{H}\left( t-\tau\right) \right]N_{i}^{R}d\tau$ | (A1) |
| --- | --- | --- |
|  | $I_{ij}^{C}\left( t \right)=\beta\int_{o}^{t} e^{-\gamma\tau}\left[ I_{i}^{R}\left( t-\tau\right)+I_{i}^{H}\left( t-\tau\right)+I_{j}^{W}\left( t-\tau\right) \right]N_{ij}^{C}d\tau$ | (A2) |

Here, $I_{i}^{H}\left( t \right)\equiv\sum_{j} I_{ij}^{C}\left( t \right)$and $I_{j}^{W}\left( t \right)\equiv\sum_{i} I_{ij}^{C}\left( t \right)$denote the number of infectious commuters in *i*-th home population and in *j*-th work population, respectively. By summing over indices *i* and *j* of Eq. (A2) and introducing a vector notation for non-commuting resident population ($\boldsymbol{I}^{\boldsymbol{R}}\left( t \right)\equiv\left( I_{1}^{R}\left( t \right),I_{2}^{R}\left( t \right), \cdots I_{M}^{R}\left( t \right) \right)^{t}$), commuting home population ($\boldsymbol{I}^{\boldsymbol{H}}\left( t \right)\equiv\left( I_{1}^{H}\left( t \right),I_{2}^{H}\left( t \right), \cdots I_{M}^{H}\left( t \right) \right)^{t}$), and commuting work population ($\boldsymbol{I}^{\boldsymbol{W}}\left( t \right)\equiv\left( I_{1}^{W}\left( t \right),I_{2}^{W}\left( t \right), \cdots I_{M}^{W}\left( t \right) \right)^{t}$), Eqs. (A1), (A2) can be summarized in the 3 × 3 block matrix form as

|  | $\left[ \begin{matrix} \boldsymbol{I}^{\boldsymbol{R}}\left( t \right) \\ \boldsymbol{I}^{\boldsymbol{H}}\left( t \right) \\ \boldsymbol{I}^{\boldsymbol{W}}\left( t \right) \end{matrix} \right]=\beta\int_{o}^{t} e^{-\gamma\tau}\left[ \begin{matrix} \boldsymbol{T}_{RR} & \boldsymbol{T}_{RH} & \text{0} \\ \boldsymbol{T}_{HR} & \boldsymbol{T}_{HH} & \boldsymbol{T}_{HW} \\ \boldsymbol{T}_{WR} & \boldsymbol{T}_{WH} & \boldsymbol{T}_{WW} \end{matrix} \right]\left[ \begin{matrix} \boldsymbol{I}^{\boldsymbol{R}}\left( t-\tau\right) \\ \boldsymbol{I}^{\boldsymbol{H}}\left( t-\tau\right) \\ \boldsymbol{I}^{\boldsymbol{W}}\left( t-\tau\right) \end{matrix} \right]d\tau$. | (A3) |
| --- | --- | --- |

Here, element of this block matrix $\boldsymbol{T}_{mn}$ is a $M\times M$ matrix and denotes the transmission from type *n* population to type *m* population ($m, n \in\left\{ R, H, W \right\}$, *R*: non-commuting resident population, *H*: commuting home population, *W*: commuting work population), where each element of the matrices are given as $\left[ \boldsymbol{T}_{RR} \right]_{ik}\equiv2N_{i}^{R}\delta_{ik}$, $\left[ \boldsymbol{T}_{RH} \right]_{ik}\equiv N_{i}^{R}\delta_{ik}$, $\left[ \boldsymbol{T}_{HR} \right]_{ik}\equiv N_{i}^{H}\delta_{ik}$, $\left[ \boldsymbol{T}_{HH} \right]_{ik}\equiv N_{i}^{H}\delta_{ik}$, $\left[ \boldsymbol{T}_{HW} \right]_{ik}\equiv N_{ik}$, $\left[ \boldsymbol{T}_{WR} \right]_{ik}\equiv N_{ki}$, $\left[ \boldsymbol{T}_{WH} \right]_{ik}\equiv N_{ki}$, and $\left[ \boldsymbol{T}_{WW} \right]_{ik}\equiv N_{i}^{W}\delta_{ik}$ ($\boldsymbol{0}$: $M\times M$ zero matrix). Here, it should be noted that the matrix $\boldsymbol{T}_{mn}$ includes the information about the host population structure only and the epidemiological information is not included. Since $\beta\int_{o}^{t} e^{-\gamma\tau}\boldsymbol{T}_{mn}\boldsymbol{I}^{\boldsymbol{n}}\left( t-\tau\right)d\tau$gives the number of infectious individual from type *n* population to type *m* population, the asymptotic ratio between these population types at exponential growth phase are given as $\beta\int_{o}^{\infty} e^{-\gamma\tau}\boldsymbol{T}_{mn}d\tau=\beta/\gamma\boldsymbol{T}_{mn}$. Given this the next generation matrix $\boldsymbol{L}$ is given as a $3\times3$ block matrix form as

|  | $\boldsymbol{L}=\beta\int_{o}^{\infty} e^{-\gamma\tau}\left[ \begin{matrix} \boldsymbol{T}_{RR} & \boldsymbol{T}_{RH} & \text{0} \\ \boldsymbol{T}_{HR} & \boldsymbol{T}_{HH} & \boldsymbol{T}_{HW} \\ \boldsymbol{T}_{WR} & \boldsymbol{T}_{WH} & \boldsymbol{T}_{WW} \end{matrix} \right]d\tau=\frac{\beta}{\gamma}\left[ \begin{matrix} \boldsymbol{T}_{RR} & \boldsymbol{T}_{RH} & \text{0} \\ \boldsymbol{T}_{HR} & \boldsymbol{T}_{HH} & \boldsymbol{T}_{HW} \\ \boldsymbol{T}_{WR} & \boldsymbol{T}_{WH} & \boldsymbol{T}_{WW} \end{matrix} \right].$ | (A4) |
| --- | --- | --- |

Here, each element of the block matrix gives the asymptotic ratio between different population types. Accordingly, the dominant eigenvalue of this next generation matrix $\boldsymbol{L}$ gives the basic reproductive ratio $R_{0}\boldsymbol{=}\rho\left( \boldsymbol{L} \right)$ ($\rho\left( \cdots\right)$: spectral radius).

For the dominant eigenvalue $R_{0}$, the elements of corresponding left and right eigenvector give the reproductive value and the relative ratio of exponentially growing infected population, respectively [1–3]. The reproductive value (i.e., element of left eigenvector $\left( v_{i}^{R}, v_{i}^{H}, v_{i}^{W} \right)$) of each local population is given in S2 Fig A as a function of its local population size $\left( N_{i}^{R}, N_{i}^{H}, N_{i}^{W} \right)$. The relative ratio of exponentially growing infected population (i.e., element of right eigenvector $\left( w_{i}^{R}, w_{i}^{H}, w_{i}^{W} \right)$) of each local population is given in S2 Fig B as a function of its local population size $\left( N_{i}^{R}, N_{i}^{H}, N_{i}^{W} \right)$. Both of the values increase as the local population size increase. This means that, a local population with a larger population size has a larger impact on the overall epidemic dynamics and also has a higher risk of infection. Furthermore, the most notable point is that, the results can be clustered into two distinct groups. This separation can be explained in the relation to the largest work population (i.e., working/studying area of Shinjuku station). For the results of non-commuting resident population and commuting home population (S2 Fig A2 and A3, S2 Fig B2 and B3), the local populations in the upper cluster has at least one commuter who is working/studying at the largest work population. The horizontally layered colored structure can be clearly explained by the number of commuters to the largest work population. On the other hand, no one from the local populations in the lower cluster is working/studying at the largest work population. For the results of commuting population at work population (S2 Fig A1, S2 Fig B1), the upper cluster is the largest work population itself and the lower cluster is consisted from other local populations. The clear distinction observed in the change in the basic reproductive ratio ${\delta R}_{0}$ (see Fig 3), can be attributed to this distinction in the eigenvectors.

1. Diekmann O, Heesterbeek JAP. Mathematical Epidemiology of Infectious Diseases: Model Building, Analysis and Interpretation (Wiley Series in Mathematical & Computational Biology). JOHN WILEY & SON; 2000.

2. Caswell H. Matrix Population Models. Second. Sinauer Associates Inc.; 2001.

3. Ellner SP, Guckenheimer J. Dynamic Models in Biology. Princeton University Press; 2011.
