# Supplementary material for: Spotting Epidemic Keystones by R0 Sensitivity Analysis: High-Risk Stations in the Tokyo Metropolitan Area
Source: PLoS One. 2016 Sep 8;11(9):e0162406. doi: 10.1371/journal.pone.0162406 (PMC5015857; doi:10.1371/journal.pone.0162406)
Supplement: S2 File — The definition of the final size of epidemic and the derivation of the final size equation are given. (DOCX) [file pone.0162406.s009.docx]

**S2 File. Final size of epidemic**

Overall damage can be evaluated by the global final size of epidemic $\Psi$, which is defined as a ratio of infected individuals whom has ever acquired infection during the epidemic period. For this we define the local final size of epidemic $\Psi_{i}^{R}$ within the non-commuting resident population at *i*-th station as

|  | $\Psi_{i}^{R}\equiv\frac{R_{i}^{R}\left( \infty\right)}{N_{i}^{R}}=\frac{N_{i}^{R}-S_{i}^{R}\left( \infty\right)}{N_{i}^{R}}\cong1-\frac{S_{i}^{R}\left( \infty\right)}{S_{i}^{R}\left( 0 \right)},$ | (A1) |
| --- | --- | --- |

and the local final size of epidemic $\Psi_{ij}^{C}$ within the commuting population consisted from a commuters residing at *i*-th home population and working at *j*-th work population as

|  | $\Psi_{ij}^{C}\equiv\frac{R_{ij}^{C}\left( \infty\right)}{N_{ij}^{C}}=\frac{N_{ij}^{C}-S_{ij}^{C}\left( \infty\right)}{N_{ij}^{C}}\cong1-\frac{S_{ij}^{C}\left( \infty\right)}{S_{ij}^{C}\left( 0 \right)}.$ | (A2) |
| --- | --- | --- |

Here, we have used the approximations $S_{i}^{R}\left( 0 \right)\cong N_{i}^{R}$ and $S_{ij}^{C}\left( 0 \right)\cong N_{ij}^{C}$. These can be calculated in the following way. The integral forms of Eqs. (1), (4) and Eqs. (3), (6) are given as following.

|  | $S_{i}^{R}\left( t \right)=S_{i}^{R}\left( 0 \right)\text{ exp}\left\{ -\beta\int_{o}^{t} \left[ 2I_{i}^{R}\left( s \right)+\sum_{k} I_{ik}^{C}\left( s \right) \right]ds \right\}$ | (A3) |
| --- | --- | --- |
|  | $S_{ij}^{C}\left( t \right)=S_{ij}^{C}\left( 0 \right)\text{ exp}\left\{ -\beta\int_{o}^{t} \left[ I_{i}^{R}\left( s \right)+\sum_{k} \left( I_{ik}^{C}\left( s \right)+I_{kj}^{C}\left( s \right) \right) \right]ds \right\}$ | (A4) |
|  | $R_{i}^{R}\left( t \right)=\gamma\int_{o}^{t} I_{i}^{R}\left( t \right)ds$ | (A5) |
|  | $R_{ij}^{C}\left( t \right)=\gamma\int_{o}^{t} I_{ij}^{C}\left( t \right)ds$ | (A6) |

Substitution of Eqs. (A3)-(A6) with $t=\infty$ to the definition of the local final size of epidemic Eq. (A1) and Eq. (A2) yields

|  | $\Psi_{i}^{R}=1- \text{exp}\left\{ -\beta\int_{o}^{\infty} \left[ 2I_{i}^{R}\left( s \right)+\sum_{k} I_{ik}^{C}\left( s \right) \right]ds \right\}$ $=1- \text{exp}\left\{ -\frac{\beta}{\gamma}\left[ 2N_{i}^{R}\Psi_{i}^{R}+\sum_{k} N_{ik}^{C}\Psi_{ik}^{C} \right] \right\},$ | (A7) |
| --- | --- | --- |
|  | $\Psi_{ij}^{C}=1- \text{exp}\left\{ -\beta\int_{o}^{\infty} \left[ I_{i}^{R}\left( s \right)+\sum_{k} \left( I_{ik}^{C}\left( s \right)+I_{kj}^{C}\left( s \right) \right) \right]ds \right\}$ $=1- \text{exp}\left\{ -\frac{\beta}{\gamma}\left[ N_{i}^{R}\Psi_{i}^{R}+\sum_{k} \left( N_{ik}^{C}\Psi_{ik}^{C}+N_{kj}^{C}\Psi_{kj}^{C} \right) \right] \right\}.$ | (A8) |

This system of transcendental equation with $M\left( M+1 \right)\sim100,000$ equations can be solved numerically; by recursive calculation starting from $\Psi_{i}^{R}=1$ and $\Psi_{ij}^{C}=1$ until all the values converges to a fixed point. Once this value $\Psi_{ij}^{C}$ has been obtained, the local final size of epidemic for commuting population at each home population $\Psi_{i}^{H}$ and each work population $\Psi_{j}^{W}$ can be obtained in the following way.

|  | $\Psi_{i}^{H}\equiv\frac{R_{i}^{H}\left( \infty\right)}{N_{i}^{H}}=\frac{\sum_{j} N_{ij}^{C}\Psi_{ij}^{C}}{N_{i}^{H}}$ | (A9) |
| --- | --- | --- |
|  | $\Psi_{j}^{W}\equiv\frac{R_{j}^{W}\left( \infty\right)}{N_{j}^{W}}=\frac{\sum_{i} N_{ij}^{C}\Psi_{ij}^{C}}{N_{j}^{W}}$ | (A10) |

Here the number of recovered individuals at *i*-th home population (*j*-th work population) is denoted as $R_{i}^{H}\left( t \right)\equiv\sum_{j} R_{ij}^{C}\left( t \right)$ ($R_{j}^{W}\left( t \right)\equiv\sum_{i} R_{ij}^{C}\left( t \right)$). The final size of epidemic within a total non-commuting population $\Psi^{R}$ and total commuting population $\Psi^{C}$ can be obtained in similar fashion from $\Psi_{i}^{R}$ and $\Psi_{ij}^{C}$ as

|  | $\Psi^{R}=\frac{\sum_{i} R_{i}^{R}\left( \infty\right)}{\sum_{i} N_{i}^{R}}=\frac{\sum_{i} N_{i}^{R}\Psi_{i}^{R}}{N^{R}},$ | (A11) |
| --- | --- | --- |
|  | $\Psi^{C}=\frac{\sum_{i} \sum_{j} R_{ij}^{C}\left( \infty\right)}{\sum_{i} \sum_{j} N_{ij}^{C}}=\frac{\sum_{i} \sum_{j} N_{ij}^{C}\Psi_{ij}^{C}}{N^{C}}.$ | (A12) |

Then the global final size of epidemic $\Psi$ is given from $\Psi^{R}$ and $\Psi^{C}$ as

|  | $\Psi\equiv\frac{\sum_{i} R_{i}^{R}\left( \infty\right)+\sum_{i} \sum_{j} R_{ij}^{C}\left( \infty\right)}{N}=\frac{N^{R}\Psi^{R}+N^{C}\Psi^{C}}{N}.$ | (A13) |
| --- | --- | --- |

Dependence of the final size of epidemic within the non-commuting population $\Psi^{R}$, the commuting population $\Psi^{C}$ and for the total population $\Psi$ on the infection rate $\beta$ is given in S1 Fig A. For both cases of $r=0$ and $r=1$, when the infection rate is small, the final sizes of epidemic are negligible such that the initial extinction of disease occurs, however as the infection rate increases the global final sizes of epidemic monotonically increase until it saturates to one. The infection rates at these disease invasion thresholds agree with $\beta_{c}$ obtained from the calculation of basic reproductive ratio (cf. Eq. 8). These critical infection rate $\beta_{c}$ was slightly larger for $r=0$ ($\beta_{c}=9.210485\times{10}^{-5}$) compare to $r=1$ ($\beta_{c}=9.207523\times{10}^{-5}$), this can be attributed to the larger total population size for $r=1$. This fact suggests that the effect of non-commuting population is minimal for the disease invasion condition, such that even thought the total population is doubled the effect to the threshold value $\beta_{c}$ is minimal. For $r=1$, the final size of epidemic is larger for commuting population ($\Psi^{C}$) compare to that of the non-commuting population ($\Psi^{R}$), and the disease invasion occurs from slightly smaller infection rate. These can be ascribed to the fact that the commuting individuals have a higher risk of encountering infectious individuals at the work population compare to the non-commuting residents. In such way, there is a quantitative difference between the results of $r=0$ and $r=1$. However, qualitative differences are minor, therefore, throughout this study we have used $r=1$ in the analysis.
